# Multi-Omics integration can be used to rescue metabolic information for some of the dark region of the *Pseudomonas putida* proteome

**DOI:** 10.1186/s12864-024-10082-y

**Published:** 2024-03-11

**Authors:** Steven Tavis, Robert L. Hettich

**Affiliations:** 1 Genome Science and Technology Graduate Program, University of Tennessee Knoxville, Knoxville, USA; 2 Biosciences Division, Oak Ridge National Laboratory, Oak Ridge, TN USA

**Keywords:** Multi-omics integration, Proteins of unknown function, Machine learning, Gene ontology, Pseudomonas putida, Function prediction

## Abstract

In every omics experiment, genes or their products are identified for which even state of the art tools are unable to assign a function. In the biotechnology chassis organism *Pseudomonas putida,* these proteins of unknown function make up 14% of the proteome. This missing information can bias analyses since these proteins can carry out functions which impact the engineering of organisms. As a consequence of predicting protein function across all organisms, function prediction tools generally fail to use all of the types of data available for any specific organism, including protein and transcript expression information. Additionally, the release of Alphafold predictions for all Uniprot proteins provides a novel opportunity for leveraging structural information. We constructed a bespoke machine learning model to predict the function of recalcitrant proteins of unknown function in *Pseudomonas putida* based on these sources of data, which annotated 1079 terms to 213 proteins. Among the predicted functions supplied by the model, we found evidence for a significant overrepresentation of nitrogen metabolism and macromolecule processing proteins. These findings were corroborated by manual analyses of selected proteins which identified, among others, a functionally unannotated operon that likely encodes a branch of the shikimate pathway.

**Supplementary Information:**

The online version contains supplementary material available at 10.1186/s12864-024-10082-y.

## Introduction

*Pseudomonas putida* is a promising chassis bacterium that is being customized and deployed for a range of biotechnology applications [1], including lignin valorization [2, 3] and the production of biofuels [3]. These applications necessarily involve engineering the genome of *P. putida* in order to produce novel metabolites and optimize the synthesis of natural products. Critical to bioengineering projects of this nature is a complete understanding of the enzymes, transporters, and regulatory systems involved in a pathway of interest. Proteomics measurements of *P. putida* routinely identify the differential expression of not only numerous annotated proteins but also proteins of unknown function (PUFs), including in conditions highly relevant to biotechnological applications. It is probable that some, and possible that many of these proteins, are nonfunctional or unexpressed pseudogenes. However, pseudogenes rarely make up more than 5% of bacterial genomes [4] and are likely to be under negative selection [5] so it is likely that most PUFs are functionally relevant. This terra incognita of the proteome is by necessity routinely ignored in proteomics analysis but represents a dangerous blind spot in our understanding and control of the functional genetics of *P. putida*.

There is a remarkable diversity of functions carried out by proteins, which has led to the creation of multiple standardized systems for describing protein function in a computationally approachable manner [6, 7], of which the most popular is the Gene Ontology [8, 9]. Such standardized and structured function labels are necessary for the omics-scale analysis of expression data, as it brings prior knowledge to bear on such analyses, reduces their severe multiplicity, and facilitates cross-species comparisons [10].

Current approaches to function annotation largely rely on inferences based on sequence similarity between genes in different organisms [11]. Traditional approaches were based on the simple transfer of annotations from high scoring BLAST hits [12]. Subsequent methods used hidden Markov models and other sequence pattern identification tools to identify domains and higher order family relationships [13]. There have been numerous successful efforts to integrate other sources of information, including evolutionary relationships [14, 15], protein–protein interaction networks [16], co-expression data [17, 18], and text mining [16, 19]. In the most recently published Critical Assessment of Function Annotation (CAFA3) challenge [20], most models incorporated some amount of non-sequence similarity information in their predictions, typically evolutionary relationships. The challenge highlighted the usefulness of incorporating this information and in particular identified NetGO [16] as a high-quality model that takes advantage of the STRING database of multidimensional protein–protein similarity information [21].

The best performing model in CAFA3 was overall better than CAFA2 but the improvements were neither large nor consistent across sub-tasks [20]. It may be the case that current tools are reaching the limits of what inferring function primarily from sequence similarity is capable of and that greater integration of diverse sources of information is necessary. A critical weakness of general-purpose function prediction approaches is that they, by necessity, are not tailored to the information available for a specific organism. NetGO uses the STRING database to combat this issue, which allows the tool to take advantage of a collection of databases of targeted experiments. However, for many organisms, *P. putida* included, these databases have very limited information.

Unfortunately, there is no organism for which the functional annotation of its genome is complete [22]. The presence of PUFs in differential expression experiments, especially differentially abundant PUFs, brings a risk of bias in subsequent gene ontology enrichment analyses; this is because the differential abundance associated with a GO term is compared against the background rate of expression for that term. If PUFs are present in the dataset, they can bias the estimates of GO frequencies in either the background or differentially abundant protein sets. The presence of PUFs also drive a spotlight effect wherein analyses focus on the functions which are known to be present and the functions of PUFs are ignored.

A previous work on predicting the function of recalcitrant PUFs focused on a minimal bacterial genome [23]. It was found that recalcitrant PUFs were strongly enriched in transporters, which was interpreted as a result of the nutrient rich environmental niche of the organism driving the need for an unusual diversity of transporters. We took a different line of thought in that we expected that proteins which are more difficult to purify and thus more difficult to biochemically assay would be systematically under–annotated regardless of organism, meaning that PUFs, at least in comparatively common laboratory bacteria such as *Pseudomonas* species, should generally be enriched in membrane and structural proteins. However, our untargeted analysis of protein function found that PUFs in *P. putida* are in fact depleted in these functions.

We focus our analysis on the proteins which completely lack annotation because, compared to partially annotated proteins, they present a greater risk of bias in GO enrichment analyses and they provide no starting point for hypothesis driven experimental assessment of function. Even partial or shallow information is valuable in these cases. We are particularly concerned with the general categories of functions that are enriched in recalcitrant PUFs, as this allows us to assess the importance of this class of proteins for biotechnologists.

This work seeks to extend the state-of-the-art automatic GO annotations of *P. putida* by constructing a bespoke predictive model that is tailored to the datasets available for the organism. To interrogate the function of recalcitrant PUFs while maintaining control of the false discovery rate (FDR) of annotations, we integrate popularly used lines of evidence. These include evolutionary analysis, online databases, sequence and structural similarities, and co-expression data. Different lines of evidence can be used to assess intra- and inter-species protein similarity, so a two-part model is built with one arm using within species proteome-scale data and the other leveraging Alphafold [24, 25] structural predictions to identify cross-species structural similarities. We find that PUFs carry out a quantitatively distinct distribution of functions and are enriched in both nitrogen metabolism and metabolite binding.

## Materials and methods

### Genome data

Unless otherwise specified, all analyses used the genome downloaded as a.faa file from the Pseudomonas Genome Database for strain KT2440_110. All Uniprot and Alphafold data are for NCBI:txid160488.

### Initial function annotations

Gene Ontology (GO) functional annotations were downloaded from Biocyc [26], The Pseudomonas Genome Database [27], and Uniprot [28] on Oct. 22, 2020. All annotation files were for the KT2440 strain. Evidence code summaries are listed in Table S1 for Biocyc, Table S2 for the Pseudomonas Genome Database, and Table S3 for Uniprot; all are found in Additional file 3. Further annotations were obtained using the online tool NetGO2.0. NetGO annotations were filtered to have a quality score greater than 0.9, which appeared to be an inflection point in the cumulative score distribution and was thus considered the likely limit of high confidence predictions. Proteins were considered to be PUFs if they were annotated to a depth of less than two, meaning no annotation or only annotated with an uninformative ontology root.

#### Similarity of sequence motifs

InterProScan 5 [13] was run locally on all proteins using default settings. SignalP 5.0 [29] was also run on these proteins with default settings. A binary presence-absence vector was made for each protein to represent the sequence features assigned to it. The Shannon information content of each feature in the dataset was calculated and used as a weight for that feature. For all pairwise comparisons of proteins, the weighted Jaccard distance of their feature vectors was calculated and used as a predictor.

#### Sequence similarity

Diamond [30] was used to assess the sequence similarity of *P. putida* proteins. All PUFs were searched against the entire *P. putida* genome database with ultra-sensitive mode enabled. The bitscore of the alignments were used as the similarity measure.

#### Operon co-membership

**Fig. 1:**
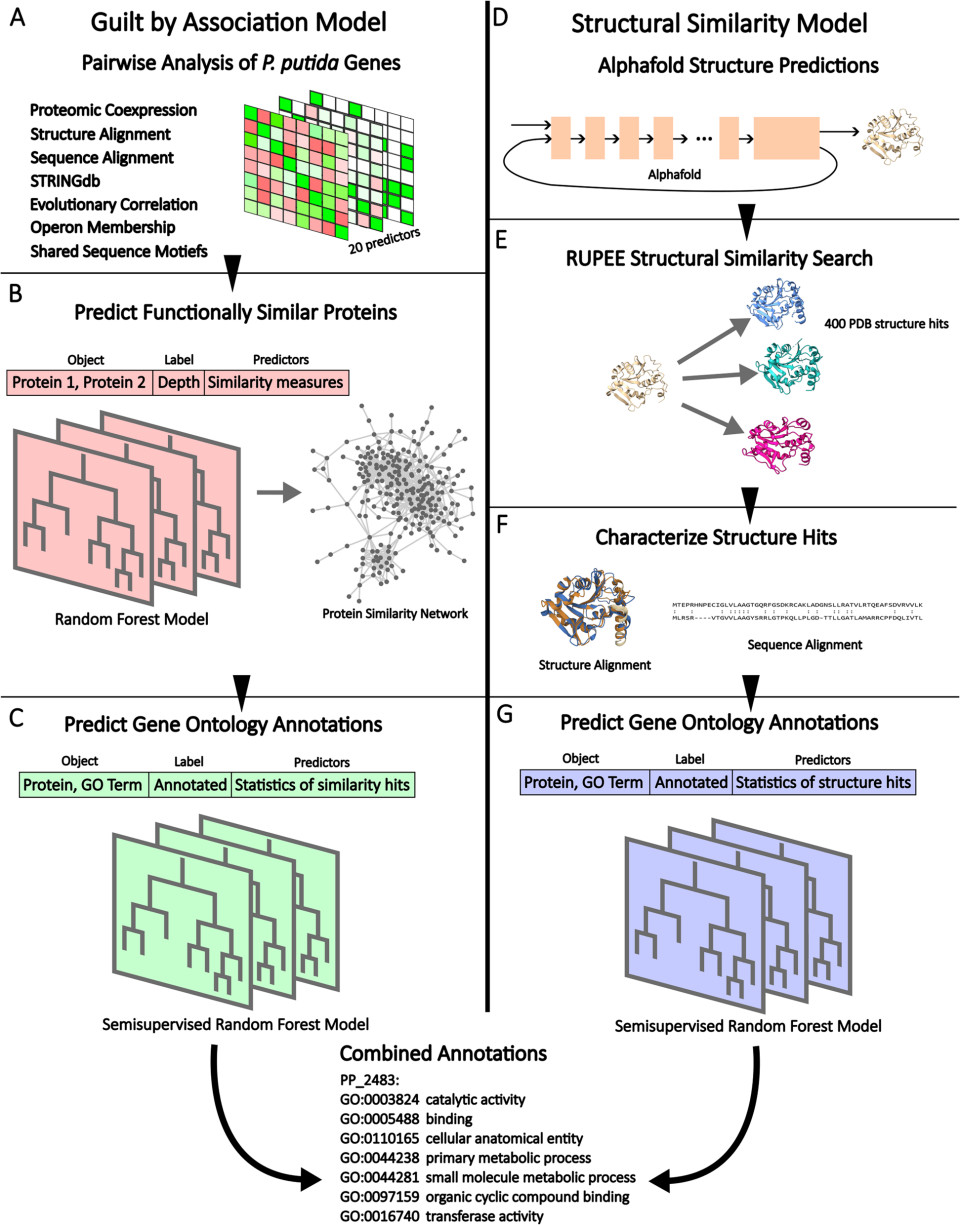
**A**-**C** Within species guilt-by-association predictive model. **D**-**G** Between species structural similarity predictive model. **A** 20 measures of protein similarity are calculated for all pairs of *P. putida* proteins. **B** The depth of deepest shared GO term is predicted for each pair of proteins based on the 20 measures of similarity. Hits are considered to have a depth > 6. **C** The matching of a term to a protein is predicted from summary statistics of hits that contain the term. **D** Alphafold structure predictions are downloaded from the Alphafold database. **E** PUF structures and a matching number of PKF structures are searched against the PDB using RUPEE. **F** Predictors are calculated from RUPEE structure alignments and NWalign sequence alignments. **G** Annotation matches are predicted as in (**C**) from hits annotated with each term. Final annotations are the union of the output of the two models

Transcriptomics data were downloaded from the JGI genome portal for JGI project ID 1137772. Rockhopper [31] was then used to identify operons by mapping transcripts to the genome using default settings. Co-membership was included in the within species protein–protein similarity model (Fig. 1B) as a binary predictor variable.

#### Evolutionary correlations

The amino acid sequences for 612 complete genomes hosted by the Pseudomonas Genome Database were downloaded as fasta files and associated gff3 on Mar. 9, 2021. Groups of orthologs were identified with proteinortho [32] using default parameters and the additional synteny parameter. The size of the intersection of species sets in these orthogroups was calculated for all pairwise comparisons of *P. putida* proteins. Multisequence alignments of orthogroups containing *P. putida* genes were generated using MAFFT [33] with automatic parameters. Phylogenetic trees for each orthogroup were estimated using RAxML-ng [34] with default parameters. A species tree was estimated from these gene trees using Astral-III [35] with default parameters. Gene tree branch lengths were then re-estimated using RAxML-ng with default parameters. Robinson-Foulds weighted cluster metric was calculated for all pairwise comparisons of trees using TreeCMP [36] with parameters to prune unmatched species and allow for 0 length branches. Both the orthogroup intersection size and the Robinson-Foulds weighted cluster metric were then used as predictors in the within species protein–protein similarity model (Fig. 1B).

#### Proteomic co-expression

ProteomeXchange [37] was searched for bottom up proteomics datasets containing only *P. putida* proteins. Datasets were restricted to label free DDA data with at least five conditions to enable combined processing and to ensure that correlations were stable within each dataset. Datasets were also excluded if files could not be mapped to samples due to poor metadata. Raw files for three proteomics datasets (ProteomeXchange identifiers: PXD013011, PXD016028, and PXD016114) passed these requirements with 8, 18, and 12 conditions respectively. Throughout the process, each experiment was processed separately with identical parameters. Our data processing pipeline was inspired by the results in [38]. Raw files were converted to mzML and MGF files with MSconvert [39] using ThermoFisher’s peak picking algorithm for centroiding profile mode data and otherwise default parameters. Within the Philosopher pipeline [40], runs were searched with both Comet and MSfragger with a parent ion tolerance of 10 ppm, a fragment ion tolerance of 0.02 Da and trypsin as the enzyme; all other parameters were left default. Search outputs were filtered using PeptideProphet with the accmass, decoy, and nonparam flags set and trypsin as the enzyme. iProphet was used to combine the outputs of the searches with default parameters. A spectral library was constructed from the combined search results using SpectraST with the cIHCD, cAC, cu, c_DIS, c_BDU, and c_BDT flags set. The mouse spectral library was downloaded from NIST and processed using the cAC, c_DIS!, c_BDU, and c_BDT flags set and “DECOY_” added to each entry. These two spectral libraries were then concatenated. MGF files were then searched using Ann-SoLo [41] with a precursor tolerance of 20 ppm, a precursor open mass tolerance of 500 Da, a fragment mass tolerance of 0.02 Da, peak shifts allowed, and the concatenated spectral library as the database. In a custom script, the Ann-SoLo output was FDR controlled at 1% using the mouse spectra as decoys. Peaks in the delta mass histogram with an error greater than 5 Da were treated as potential modifications. Potential modifications were filtered to identify plausible chemical artifacts; biological PTMs and PSMs in the delta mass peaks that survived this filter were annotated with the corresponding modification. Proteins were quantified using FlashLFQ [42] with both match between runs and shared peptide quantification enabled and otherwise default parameters. Nonzero intensities were averaged per condition and missing values at the condition level were zero imputed. The Spearman correlation coefficient was calculated for all pairwise comparisons of proteins on a per experiment basis, with missing proteins being given zero correlations, and the correlations were averaged across experiments. The Jaccard distance was calculated on the presence/absence vector of all conditions in all experiments. Both the mean Spearman correlation coefficient and Jaccard distance were used as predictors.

#### Structural similarity

*P. putida* protein structure predictions were downloaded from the Alphafold database [24, 25]. For each prediction, residues were trimmed starting at both termini until the first residue with a pLDDT, a confidence score predicted by Alphafold based on the work in [43, 44], greater than 70 was reached. If the untrimmed region was longer than 30 amino acids the structure was kept for downstream analysis. 3131 structures remained after this process. TM align [45] was used to calculate TM-scores, a global structure alignment quality score [46], and root mean squared deviation (RMSDs) for all pairs of proteins. Two TM-scores were calculated for each pair by normalizing against the length of each protein. The maximum of the two scores was used, along with RMSD, as similarity measures.

#### STRINGdb data

The full list of protein–protein similarity scores for *P. putida,* including sub scores, was downloaded from STRINGdb [21]. The combined score and all sub scores were used with the exception of co-expression and experiments (although co-expression transferred and experiments transferred were both used) these sub scores were excluded on the basis of the extremely low number of links they contained.

### Guilt-by-association model

The protein–protein similarity scores described above were used to predict the functional similarity of pairs of proteins (Fig. 1B). To define similarity for all possible pairs of proteins, we took the set of GO terms annotated to both and found the deepest shared term, meaning the term with the most steps in the longest path from the ontology root to the term. This number of steps was used as the measure of protein similarity. We treated GO terms from each of the three ontologies equivalently, as we found that each ontology had a qualitatively similar relationship between term depth and functional informativeness. Ten percent of annotated proteins were held out as a test dataset for all machine learning steps in the model (Figs. 1B and C). A random forest regression model was trained to predict similarity (Fig. 1B). The vector of predictors for this model is listed in Table S4. To account for unbalance in the training data, protein pairs with a shared term depth greater than six were oversampled twofold and those with a depth greater than eight were oversampled fourfold. The protein pairs with a predicted shared term depth greater than six were considered similar and used as inputs for the annotation model. The cutoff depth of six was chosen in order to balance the number of annotated proteins that could contribute information with the specificity of the information contributed to each query protein.

For the annotation model (Fig. 1C), a semi-supervised random forest classifier was trained to predict, on a term-by-term basis, whether a GO term is associated with a query protein. The predictors for this model were derived from the set of proteins identified as similar to the query by the first model, which were also annotated with the GO term being tested. Each predictor was a summary statistic describing the collection of values for one similarity measure among hit proteins. So, for a particular GO term-protein pair that we wish to test with the model, we find all of the predicted similar proteins that are annotated with that term and then summarize the similarity scores between those proteins and the query protein. For example, the sum and the maximum are used to summarize TM-scores and both values are included in the vector of predictors. The specific similarity measures and the summary statistics which form the predictive features for the model are listed in Table S5. The association of a GO term with a protein was only assessed if the term appeared among the annotations for similar proteins. Term-protein pairs were left unlabeled if the protein was in the test set, if the protein was a PUF, or if the term was deeper than the deepest term in the proteins known annotation. Preliminary testing showed that the FDR control procedure overfit when run on the training data, so an additional random 10% of the term-protein pairs from the training dataset were treated as unlabeled for use by the FDR control procedure. The model produces a continuous confidence score for each term-protein pair; a threshold was set on this score for annotating a protein with a term that resulted in a 1% FDR in the set left unlabeled for this purpose. A term passing this threshold, along with all its parent terms that were necessitated by the structure of the ontology, were predicted to be annotated to the query protein. The function predictions made by the model are available in Additional file 1.

### Network modularity analysis

In addition to their use in the annotation model (Fig. 1C), the identified protein pairs from the protein–protein similarity model (Fig. 1B) were treated as a network. We assessed binary partitions of this network, meaning disjoint sets of nodes, i.e. PUFs vs PKFs, random sets, or proteins sharing a GO term vs those not annotated with the term. The modularity score of these partitions was calculated using the modularity function provided by the NetworkX package in python [47]. The resolution value, a tunable parameter related to the characteristic size of communities within a network, was set at 0.81 using the procedure published in [48]. Random partitions were generated to be the same size as PUFs and GO terms were selected to produce partitions if they were annotated to more than 400 proteins.

### Protein structure database

Alphafold structure predictions for all proteins in Swissprot were downloaded from the Alphafold protein structure database on Aug. 2, 2022. These proteins were trimmed and filtered in the same manner as the *P. putida* structures (Fig. 1D).

### RUPEE structural similarity search

A RUPEE [49] was used to identify structurally similar proteins in the PDB database [50, 51] for all *P. putida* proteins with cleaned structure predictions. Search type was set to full length and search mode was set to all aligned. Hits with a TM-score greater than 0.3 were retained for downstream analysis (Fig. 1E).

### Sequence analysis of RUPEE hits

Amino acid sequences for each *P. putida* query protein were compared to each of its Swissprot hits using NWalign to generate additional predictors for the structural similarity model. From the outputs percent sequence identity and percent non-gap were used as predictors. Additionally, the rank order of the most specific shared taxonomic level was used as a crude measure of phylogenetic similarity i.e., genus = 1, family = 2, etc. (Fig. 1F).

### Structural similarity model

The design of the semi-supervised random forest classifier that predicts GO annotations for PUFs based on structural similarity hits (Fig. 1G) is similar to its counterpart in the guilt-by-association model (Fig. 1C). In this case, the hit proteins come from the RUPEE structural similarity searches and the predictors are summary statistics of the NWalign results and the TM-score and RMSD reported by RUPEE. The specific summary statistics used for each similarity score to construct the feature vector are listed in Table S6. Training data were again considered unlabeled if the term was deeper than the deepest annotation for the training protein, if the term-protein pair was a member of the random 10% used for FDR control, if the protein was a PUF, or if the protein was a member of the test set. Final predicted annotations were the union of predictions from both the guilt-by-association and structural similarity arms. The function predictions made by the model are available in Additional file 2.

### Analysis methods

Bayesian statistical models were written in Stan [52]. Bootstrap models were made in Python using Numpy [53]. All in house scripts used in the analysis are available on GitHub at https://github.com/stavis1/Pputida_PUF_predictions_paper (https://doi.org/10.5281/zenodo.10493789).

## Results

**Fig. 2:**
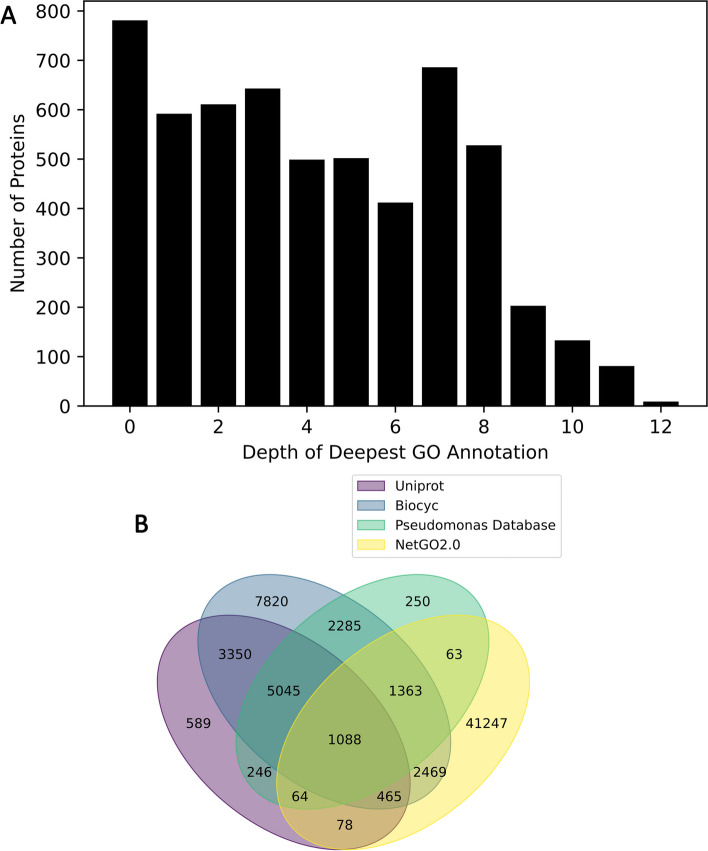
**A** Distribution of GO term depths in the initial set of annotations. **B** The number of GO terms contributed to the initial annotations by each source

State of the art functional annotations for the *P. putida* genome are incomplete and inconsistent between sources. Approximately 14% of the *P. putida* proteome are PUFs and 50% of proteins are annotated to a GO depth of less than 6 (Fig. 2B). Over 75% of these annotated terms are observed in only one of the four sources of annotations, with the majority of these singletons coming from NetGO2.0. Only 2% of annotations were observed in all four datasets, although the intersection would be 24% in the absence of NetGO2.0 predictions (Fig. 2A). Among the annotations in the global intersection, 25% of them were 'DNA-binding transcription factor activity' or 'DNA binding'. 62% of annotated terms were from the biological process ontology, while 34% were from molecular function and 4% were from cellular component.

PUFs have distinct distributions of properties and are on average shorter proteins, with a median length of 118 amino acids compared to 301 for proteins of known function (PKFs) (Additional file 3 Figure S1). The median number of orthologs identified for PUFs across 612 published *Pseudomonas* genomes was 70 genes, whereas the median number of orthologs attributed to a PKF was 474 (Additional file 3 Figure S1). The median pLDDT, a per-residue measure of prediction confidence, was 84 for PUF structure predictions from Alphafold compared to 90 for PKFs. This is likely due to the lower evolutionary conservation of PUFs, as Alphafold uses residue level evolutionary correlations to predict protein structure [24]. To assess the statistical significance of these observations against a null model of equal distributions, 500 bootstrap resamples of the quantile–quantile plots for each of these metrics were calculated and in all cases the y = x line, representing the null, lay entirely outside of the confidence interval (Additional file 3 Figure S1).

A random forest model (Fig. 1B) was constructed to predict the depth of the deepest shared GO term between pairs of proteins. These predictions were based on 20 similarity measures derived from proteomic co-expression data, structure and sequence alignments, STRINGdb, evolutionary correlations, operon membership and InterProScan features; see methods for details (Fig. 1A). Those protein pairs with a predicted shared depth of more than 6 were considered hits, which means that their similarity scores are used by the subsequent term transfer model (Fig. 1C). The area under the curve of the receiver operating characteristic (AUC-ROC) for the model is 0.77 (Additional file 3 Figure S2).

**Fig. 3:**
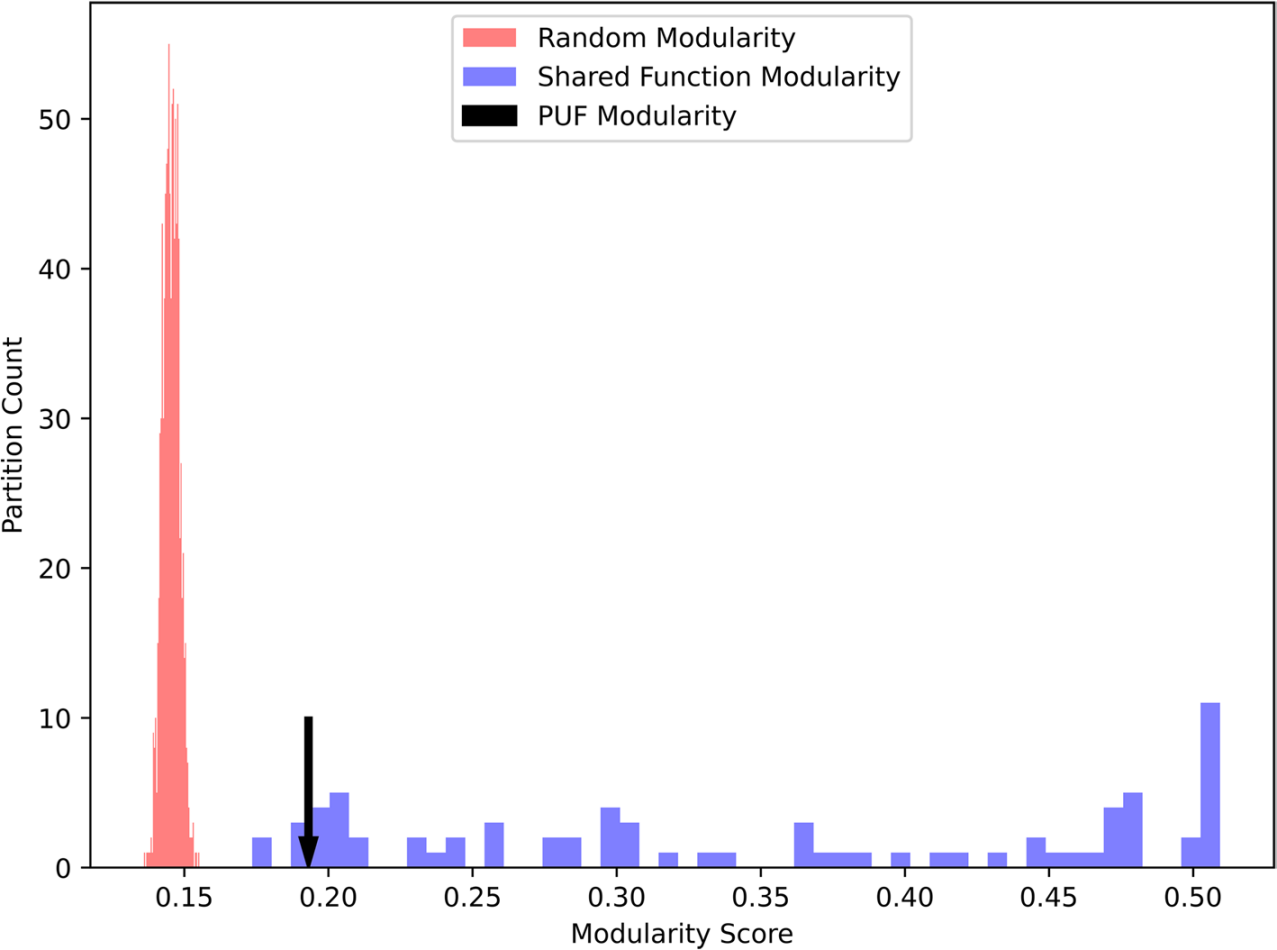
The modularity of PUFs as a partition of the protein functional similarity network compared to two models of functional coherence. The null model consists of random sets of proteins of the same size as PUFs. The shared function model consists of proteins sharing a GO term annotation, for all GO terms annotated to more than 400 proteins

Within the network of proteins made by linking the hits identified above, PUFs had a modularity score of 0.19. This number is difficult to directly interpret, so we sought to construct a conceptual scale with a model of partitioning due to chance on one end and models of partitioning due to shared function on the other. To compare this against null expectations, 1000 random partitions of the network were generated of the same size as PUFs. These partitions had uniformly lower modularity scores than PUFs, with a range of 0.14 to 0.15 (Fig. 3). By contrast the modularity of proteins sharing common GO terms ranged from 0.17 to 0.51 (Fig. 3 and Additional file 3 table S1). Of particular interest, 'oxidoreductase activity', 'intrinsic component of membrane', and 'cation binding' all had lower modularity scores than PUFs.

**Fig. 4:**
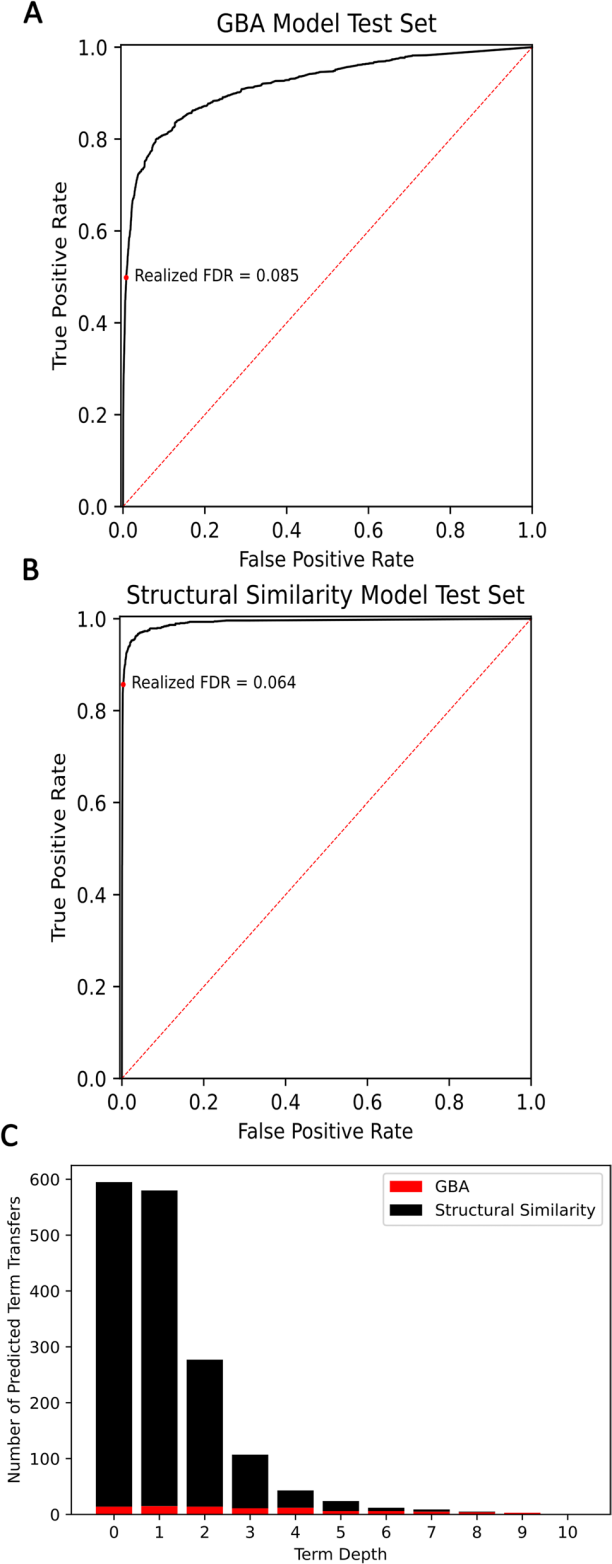
**A** ROC curve for the GBA model. Area under the curve is 0.92 and recall at the 1% FDR controlled cutoff is 0.50. **B** Receiver operating characteristic (ROC) curve for the structural similarity model. The area under the curve is 0.99 and the recall at the 1% FDR controlled cutoff is 0.86. **C** The number and depths of GO term annotations predicted by each model

Within species, protein similarities are able to extend state of the art annotations. The predicted functional similarity hits from the previously mentioned model provided the starting point for predicting GO term annotations. For each GO term found among the functional similarity hits for a PUF, we computed summary statistics of the source similarity measures of the set of hits annotated with that term (Fig. 1B, C). We predicted whether a GO term should be applied to a protein based on this vector of summary statistics. Despite the source model’s AUC-ROC of 0.77 (Additional file 3, Figure S2), the GBA model achieved an AUC-ROC of 0.92 (Fig. 4A). The score cutoff was determined by finding the cutoff which controlled the training set false discovery rate at 1%, which in the test set resulted in a realized false discovery rate of 8.5% which corresponds to a precision of 91.5%. At this cutoff, the recall in the test set is 50%. In total, 77 GO terms were able to be annotated across 17 PUFs (Fig. 4C). The full results from this model are found in Additional file 1.

The structural similarity model separately extended state of the art annotations for PUFs based on between species information transfer. This model’s construction follows the same pattern of first identifying a set of similar proteins that could provide functional information and then deciding if an annotation applied to one or more of those proteins should be transferred to the query protein based on the summary statistics of similarity scores (Fig. 1 D-G). Similar proteins here were identified using RUPEE structural similarity searching (Fig. 1E). Several similarity scores were calculated for identified pairs of proteins on the basis of sequence and structural analysis (Fig. 1F) and GO terms were transferred using a semi supervised random forest classifier (Fig. 1G). The model achieved a test set AUC-ROC of 0.99 with a recall of 86% at the 1% FDR controlled cutoff. The realized FDR at this cutoff was 6.4%, which means the precision was 93.6%. This resulted in the annotation of 1002 terms to 203 proteins (Fig. 4B and C). The full results from this model are found in Additional file 2.

**Fig. 5:**
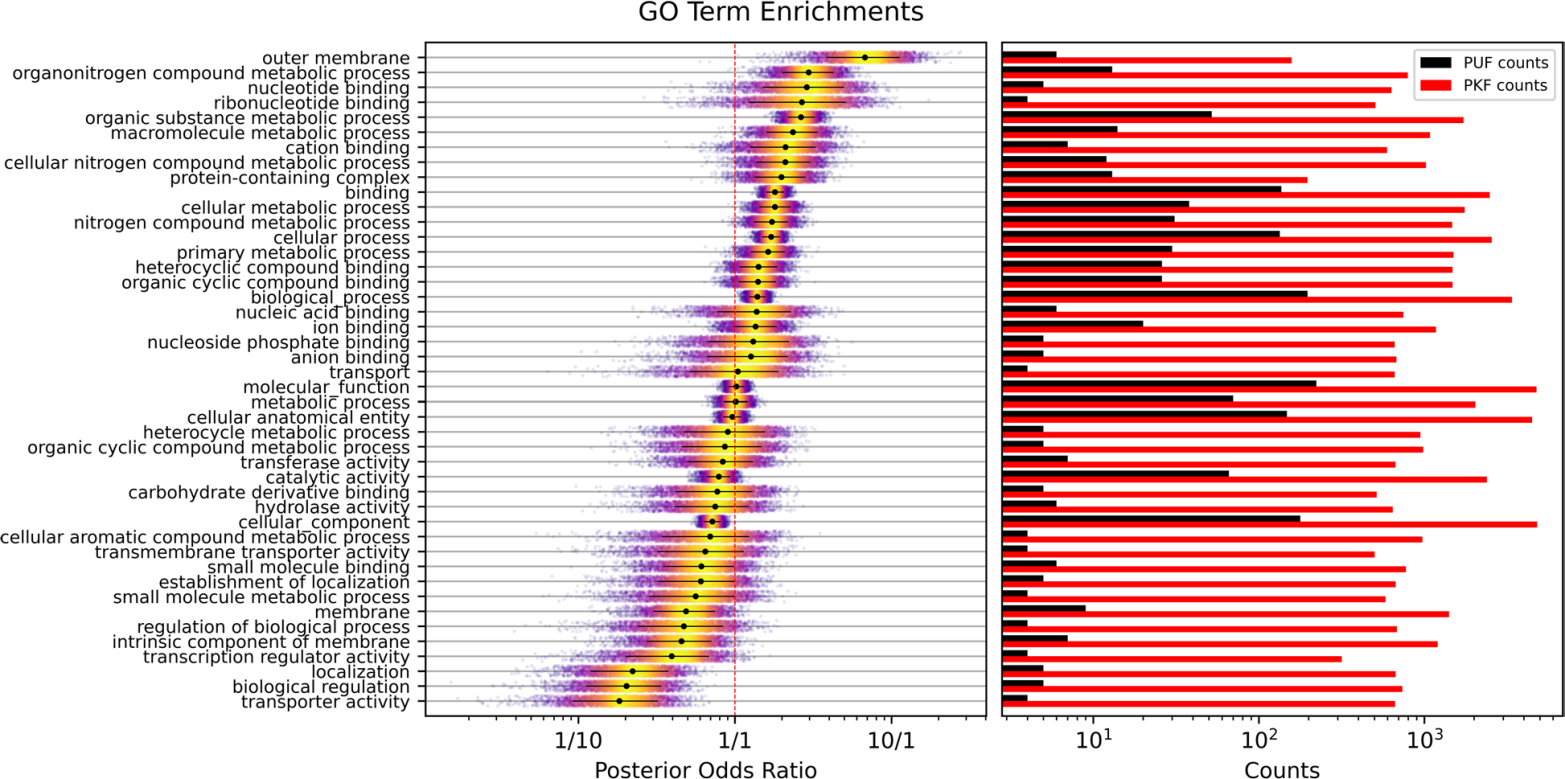
Posterior distributions of GO term enrichments quantified by PUF to PKF odds ratios. Black dots represent the medians, and lines represent 0.1–0.9 quantiles of the posterior. Color indicates posterior sample density. On the right are plotted the number of proteins annotated with each GO term

**Fig. 6:**
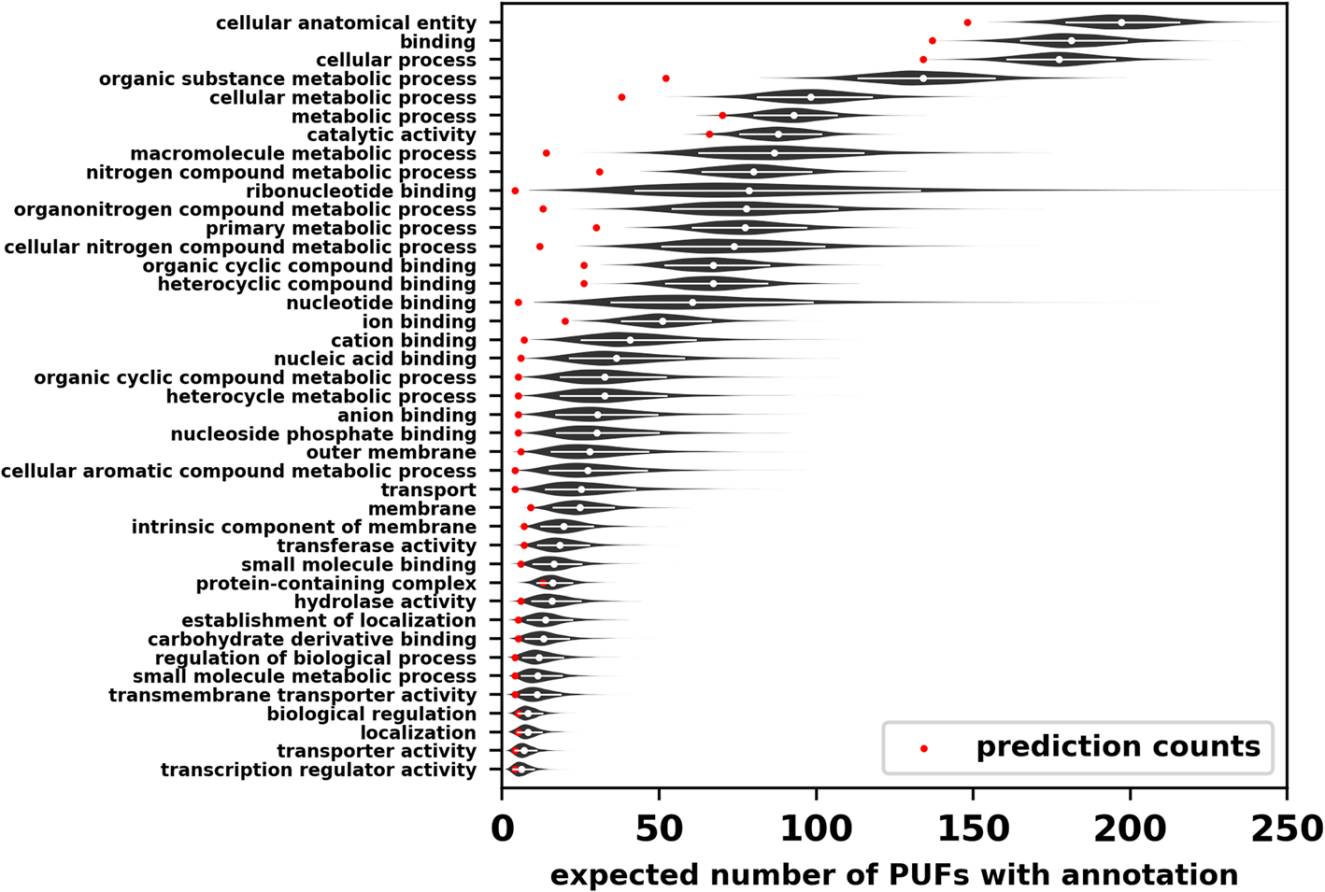
Posterior expectations for the number of PUFs annotated with a GO term if PUFs were annotated to the same completeness as PKFs. Red dots represent the number of times a term was predicted by the models. White dots represent the median, and lines represent the 0.1–0.9 quantiles of the posterior

The combined results of our two models were able to assign 1079 GO terms to 213 PUFs; the combined results are listed in Additional file 5. Using these annotations, we assessed PUFs to be enriched in metabolite binding and nitrogen metabolism. A GO overrepresentation analysis was conducted for all terms with at least four observations in both PUFs and PKFs (Fig. 5). The analysis used a Bayesian hierarchical model that accounted for the depths of predicted terms. Both strong over- and under-representations of terms were identified among the predictions, indicating that PUFs represent a quantitatively different distribution of functions than PKFs. Among the topmost overrepresented terms were numerous metabolic process terms, many of which related to nitrogen metabolism. Additionally multiple metabolite binding terms were confidently overrepresented. The three most underrepresented terms were ‘localization’, ‘biological regulation’, and ‘transporter activity’. Notably ‘membrane’ and ‘intrinsic component of membrane’ were both confidently underrepresented despite their wide posterior distributions. The most overrepresented term was ‘outer membrane’; however, this was only annotated to six PUFs. This underscores the limitation of a relative analysis for understanding the absolute functional distribution of PUFs. To understand what these results mean on an absolute scale, a second analysis was conducted to predict the total number of PUFs that should be annotated with each term if the annotations for PUFs were as complete as the annotations for PKFs. This analysis assumes that predicted annotations are an unbiased sample of true PUF annotations. A second hierarchical Bayesian model was written which takes term frequencies among PKFs as the prior and gives the expected number of PUFs based on the term frequencies among predictions (Fig. 6). The expected most common term that is functionally informative was ‘binding’ and again several metabolic process terms showed up high on the list, including ‘macromolecule metabolic process’ and ‘nitrogen compound metabolic process’. Consonant with the enrichment analysis membrane terms relating to localization, regulation, and transporters were all expected to be infrequent among PUFs.

Manual analysis of structural similarity hits can provide specific function predictions. A non-representative subset of 35 PUFs with structure predictions were selected for manual analysis based on the number of predicted terms, the quality of structure hits, and suspects from other experiments. These more detailed analyses provided more specific functional predictions than could be generated by the automatic models for 26 PUFs (Table S1, Additional file 3). Of particular note was an unannotated operon that was identified as differentially expressed in a metabolic engineering project [54, 55] (publication pending for proteomics data). Manual analysis of the collected data indicated that this operon encodes a branch of the shikimate pathway [56] which includes a chorismatase and an acetylation step.

Among the manually reanalyzed proteins are four proteins putatively involved in biofilm formation, including the pilin PP_3800, the porin PP_0576 with structural similarity to other biofilm related porins, the chaperone-usher fimbria (CsuE) like protein PP_2363, and the FapF like amyloid exporter PP_2853. The only one of these three proteins with any significant amount of information about it in the databases used in this study was PP_2363, which was referred to as a CsuE like protein by the Pseudomonas Genome Database [27]. Despite this known information, no GO annotations were obtained for this protein from any of the starting sources.

## Discussion

Proteins of unknown function, ever present in genome scale analyses, are a significant source of missing information and confounding factors in attempts to understand or control biological systems. To assess the sorts of functions hidden within *P. putida* PUFs, we constructed a two-pronged model to predict their distribution of GO terms. The first prong is a guilt-by-association model that takes advantage of within-species protein similarity measures to first predict functionally similar pairs of proteins, and then from these pairs, predict GO annotations. This produces both the predicted annotations as well as a protein functional similarity network, which is independently useful for assessing the distribution of PUF functions. The second prong leverages Alphafold structure predictions of PUFs to perform a structural analog to BLAST-based protein annotation by searching predicted structures against the PDB database of solved structures. These two prongs provide independent FDR-controlled function predictions, all of which are used for further analysis as the low overlap in outputs precluded the use of a more complicated consensus mechanism.

Based on a comparison of the two models used in this work, structural information proved to be more sensitive than sequence similarity at identifying functional similarity between proteins. Two explanations for this observation immediately present themselves: First, protein structure evolves slower than either nucleotide or amino acid sequence, which allows a structure-based search to identify more distant homologs [57]. Second, tertiary structure is a more direct driver of protein function than primary sequence, which means that structural analogs, regardless of whether they are true homologs or the result of convergent evolution, are most likely functionally informative. Structural information is also more discriminative, as proteins sharing sequence similarity but which have structural divergence, are far less likely to actually share function. The predictive power of structural analogs does have limits, as seen in the manual reanalysis of select proteins. In no example were we able to identify both the exact substrate and reaction catalyzed by an enzyme with confidence. In a particularly salient example, PP_1372 exhibited high structural similarity to a hexameric transmembrane pore involved in conjugation, a monomeric motor protein involved in DNA trafficking, and a hexameric ring DNA translocase. All of these share a degree of functional overlap but are nevertheless quite distinct. This underscores the need for a stringent quality control filter for computational function annotations, even from a source as informative as structural analogs.

The within species guilt-by-association analysis yielded far fewer confident functional predictions than the cross-species structural information. The shallow and incomplete nature of the initial annotations likely limited the amount of available information that could be used to identify protein function. This approach may be better suited to eukaryotic genomes, which are larger and more redundant so more information can be drawn upon. Nevertheless, the network of functional similarity hits was consonant with ground truth biological data in that groups of proteins all annotated with the same term generally exhibited high modularity scores. The downstream function prediction model also improved on the AUC-ROC of the similarity hit model, presumably by averaging information over multiple hits.

Both models assess the confidence with which an annotation can be applied to a query protein with a score on an arbitrary scale. A threshold needs to be set on this score in order to arrive at a final list of GO annotations for a protein. Here we set the threshold to control the false discovery rate in a holdout set of proteins. This procedure is unlikely to reliably control the false discovery rate in recalcitrant PUFs because they have a different distribution of properties. However, the realized false discovery rate in the final validation set, while higher, was still at an acceptable level for our analyses, indicating that the predicted annotations should be reliable enough to assess functional enrichment.

The results clearly indicate that the spectrum of functions performed by PUFs is meaningfully different than the functional spectrum carried out by proteins of known function. When protein–protein functional similarity predictions were treated as a network, PUFs displayed a modularity greater than expected based on their distribution of connection counts. From this observation we can reasonably assert that modularity of PUFs does represent a meaningful signal of similarity. When compared against the modularity scores calculated for sets of proteins all annotated with the same GO term, PUFs had a much lower modularity than most sets. However, 'oxidoreductase activity', 'intrinsic component of membrane', and 'cation binding' had lower modularity scores than PUFs. We take this to mean that while PUFs are certainly not a coherent functional group, they do share a noticeable degree of functional similarity, presumably due to systematic under-annotation of some functions.

The systematic difference in PUF functions compared to PKFs matters because gene ontology enrichment analyses rely on comparing function enrichments against a background. In order for this process to give unbiased estimates of the true enrichments in the face of missing annotations, those missing annotations must have the same distribution of frequencies as the known annotations. Unequal distributions can result in consistently over or underestimating the magnitude of enrichment for specific GO terms.

The hypothesis at the beginning of the project was that PUFs would be enriched in structural proteins and transporters as these classes of protein are more difficult to purify and/or assay in vitro than other classes of protein and are therefore expected to be less well annotated. However, both membrane localization terms and transporter related terms were either depleted or expected to be infrequent among PUFs (Figs. 5 and 6). GO terms related to structural functions were absent from our predictions. To our surprise, we instead identified an excess of proteins involved in metabolite binding, nitrogen metabolism, and macromolecule processing (Fig. 5). As with previous work on characterizing recalcitrant PUFs [23], this could be interpreted as the result of the environmental niche *P. putida* occupies. It has an unusually diverse capacity for catabolism, which requires an unusual number of enzymes and ancillary metabolic proteins. A diversity of proteins also means that there will be more unusual examples of proteins carrying out a specific function, which are less likely to be annotated by traditional functional annotation approaches.

The overrepresentation of macromolecule processing functions (Fig. 5) is intriguing in the context of *P. putida*’s potential utility for bioprocessing of lignocellulosic biomass. The process of lignin catabolism by *P. putida* is incompletely understood and these results point to the involvement of a significant number of PUFs. Corroborating this, 38 PUFs were found to be significantly upregulated in response to the addition of lignin feedstocks to growth media (Additional file 4). Manual analysis of these proteins identified four that are plausibly involved in biofilm formation (Additional file 3 Table S7). As there are currently only five proteins in the *P. putida* proteome that are annotated with “GO:0042710 Biofilm Formation” in the starting annotations, ignoring these PUFs changes the enrichment of this term by up to twofold. It is, of course, not the case that the community of *P. putida* researchers is only aware of five proteins involved in biofilm formation; however, automatic annotation tools, even state of the art ones, are incapable of leveraging all scientific information available for a given protein. In the context of GO enrichment analysis this sort of limited information will drive biased interpretations of omics data.

Of particular interest among the predicted annotations is the term “primary metabolic process.” With a median posterior odds ratio of 1.6, the term was only mildly enriched relative to its background frequency (Fig. 5); however, it is expected that approximately 75 PUFs would be annotated with this term were they to be annotated to the same degree of completeness as PKFs (Fig. 6). Although the term is functionally vague, it means that there are many PUFs likely involved in the metabolic processes that are continuously active. This demonstrates that understanding proteins of unknown function is critically important for metabolic engineering projects and is especially the case for projects that intersect with nitrogen metabolism, given its observed overrepresentation in PUFs.

A set of PUFs were re-analyzed by hand to produce more specific functional annotations. The limited size of this sample does not permit a stringent test of the inferences from the whole set of computational predictions; however, it does provide some corroborating evidence. Specifically, the majority of manually assessed genes were found to be enzymes, with half of them as enzymes acting on small molecule metabolites. We interpret this to mean that there is a significant unknown metabolic potential in the recalcitrant PUFs of *P. putida* that cannot be safely ignored for bioengineering or genome scale informatics. Novel pathways, alternative routes through known pathways, and the capacity to produce or consume unexplored metabolites could all reasonably exist among these proteins. For synthetic biologists this is both a challenge and an opportunity as PUFs represent both a resource to exploit and an unpredictable source of confounding factors in pathway design.

## Conclusions

Bespoke predictive modeling is able to extend state of the art function predictions for proteins of unknown function by leveraging information unique to an organism. Of particular utility in this task is the similarity of protein structures predicted by Alphafold. We predicted 1079 gene ontology terms for 213 proteins of unknown functions and analysis of these predicted functions indicates that there is a significant degree of metabolic potential among recalcitrant PUFs, especially in the areas of nitrogen metabolism and macromolecule processing.

### Supplementary Information

**Additional file 1.**

**Additional file 2.**

**Additional file 3: Figure S1.** On the left are plotted individual PUF metrics. On the right are quantile-quantile plots of the same data. White dots represent measured values black lines represent bootstrap resamples.** Figure S2.** ROC curve for the functional similarity predictive model (see Fig 1B). Area under the curve is 0.77.** Figure S3.** Results of the enrichment model for InterProScan features using the same model as the GO enrichement analysis. On the left are plotted samples from the posterior distribution of odds ratios for each element with at least 6 observations in each condition. Black dots represent the median of the posterior and black lines represent 0.1-0.9 quantiles. On the right are plotted the number of proteins annotated with each element.** Table S1.** A summary of the evidence used in Biocyc GO annotations. Count refers to the number of GO terms supported with each evidence code.** Table S2.** A summary of the evidence used in the Pseudomonas Genome Database GO annotations. Count refers to the number of GO terms supported with each evidence code.** Table S3.** A summary of the evidence used in Uniprot GO annotations. Count refers to the number of GO terms supported with each evidence code.** Table S4.** The vector of scores used in the protein-protein similarity model for the guilt-by-association arm of the analysis.** Table S5.** Summary statistics used as prediction features for the guilt-by-association annotation model.** Table S6.** Summary statistics used as prediction features for the structural similarity annotation model.** Table S7.** Hypothesized functions based on a manual analysis of the data collected for both predictive models and visualizations of RUPEE hit structural alignments using the PDB pairwise structural alignment tool.

**Additional file 4.** This file contains the notes from the manual reanalysis of specific proteins in *Pseudomonas putida*. These proteins were chosen for reanalysis based on either the large number of predicted terms from the automatic models or based on requests from collaborators.

**Additional file 5.**

## Data Availability

All data outputs are available in Additional Files included with the manuscript: Additional file 1 contains the output of the guilt-by-association model. Additional file 2 is the output of the structural similarity model. Additional file 3 contains supplementary figures and tables. Additional file 4 is the notes taken during manual reanalysis of selected proteins. Additional file 5 is the combined predicted GO terms for PUFs. In-house scripts used in the analysis are available on GitHub at https://github.com/stavis1/Pputida_PUF_predictions_paper (https://doi.org/10.5281/zenodo.8264339).
