# Supplementary material for: Multi-Omics integration can be used to rescue metabolic information for some of the dark region of the Pseudomonas putida proteome
Source: BMC Genomics. 2024 Mar 11;25:267. doi: 10.1186/s12864-024-10082-y (PMC10926591; doi:10.1186/s12864-024-10082-y)
Supplement: Supplementary file 3 — Additional file 3: Figure S1. On the left are plotted individual PUF metrics. On the right are quantile-quantile plots of the same data. White dots represent measured values black lines represent bootstrap resamples. Figure S2. ROC curve for the functional similarity predictive model (see Fig 1B). Area under the curve is 0.77. Figure S3. Results of the enrichment model for InterProScan features using the same model as the GO enrichement analysis. On the left are plotted samples from the posterior distribution of odds ratios for each element with at least 6 observations in each condition. Black dots represent the median of the posterior and black lines represent 0.1-0.9 quantiles. On the right are plotted the number of proteins annotated with each element. Table S1. A summary of the evidence used in Biocyc GO annotations. Count refers to the number of GO terms supported with each evidence code. Table S2. A summary of the evidence used in the Pseudomonas Genome Database GO annotations. Count refers to the number of GO terms supported with each evidence code. Table S3. A summary of the evidence used in Uniprot GO annotations. Count refers to the number of GO terms supported with each evidence code. Table S4. The vector of scores used in the protein-protein similarity model for the guilt-by-association arm of the analysis. Table S5. Summary statistics used as prediction features for the guilt-by-association annotation model. Table S6. Summary statistics used as prediction features for the structural similarity annotation model. Table S7. Hypothesized functions based on a manual analysis of the data collected for both predictive models and visualizations of RUPEE hit structural alignments using the PDB pairwise structural alignment tool. [file 12864_2024_10082_MOESM3_ESM.docx]

**Additional File 3. Supplementary Figures and Tables**

Figure S1


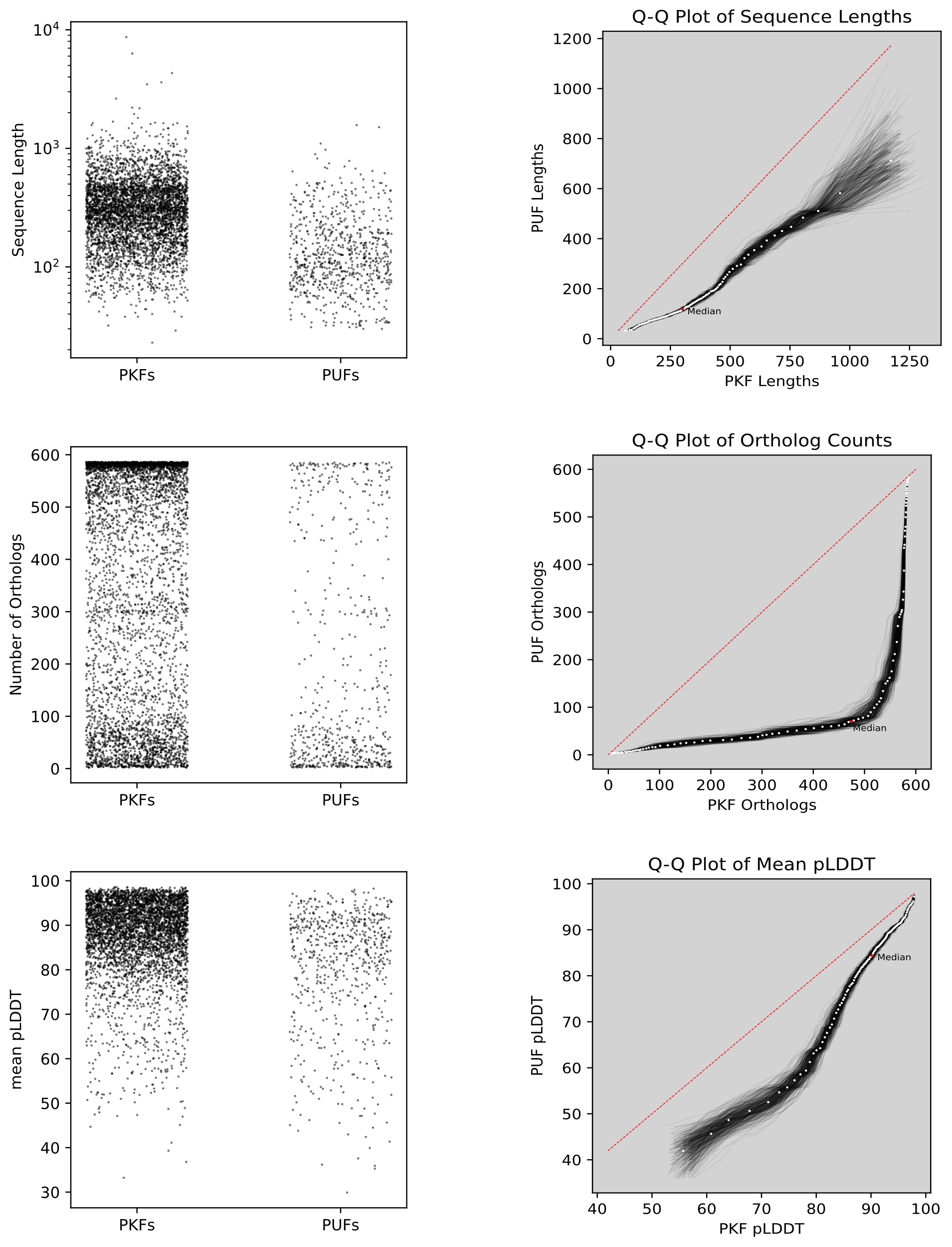


On the left are plotted individual PUF metrics. On the right are quantile-quantile plots of the same data. White dots represent measured values black lines represent bootstrap resamples.

Figure S2


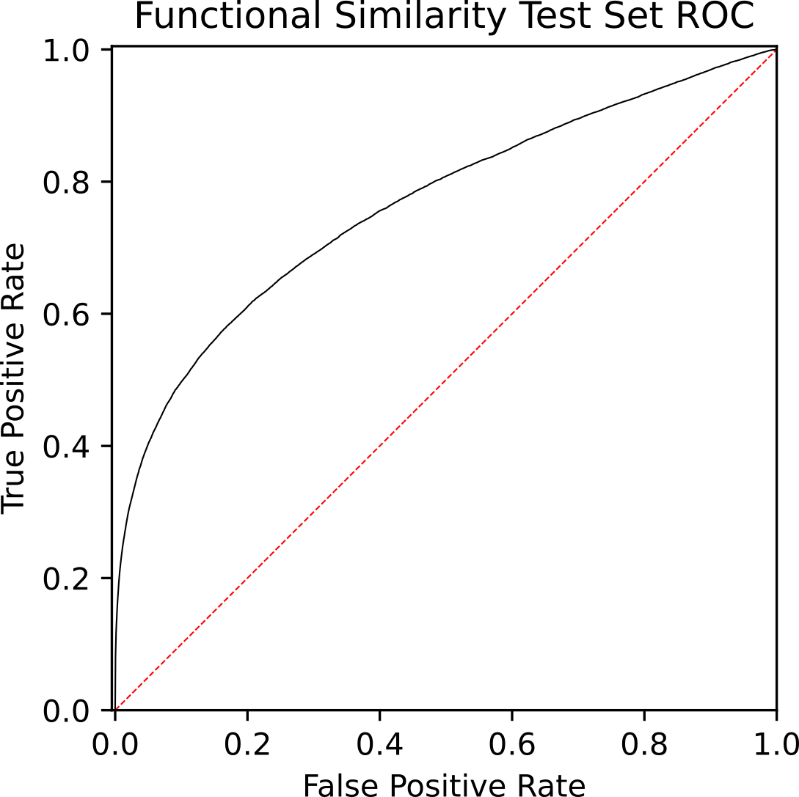


ROC curve for the functional similarity predictive model (see Fig 1B). Area under the curve is 0.77.

Figure S3


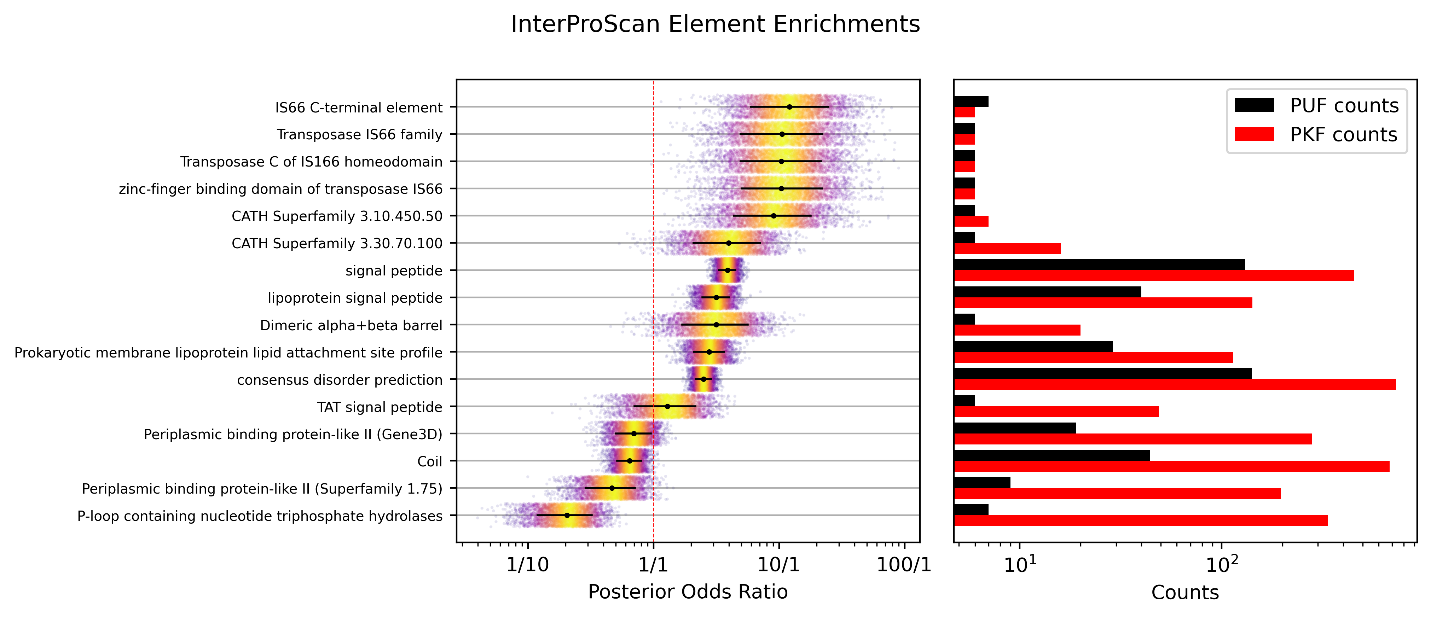


Results of the enrichment model for InterProScan features using the same model as the GO enrichement analysis. On the left are plotted samples from the posterior distribution of odds ratios for each element with at least 6 observations in each condition. Black dots represent the median of the posterior and black lines represent 0.1-0.9 quantiles. On the right are plotted the number of proteins annotated with each element.

Table S1

| GOREF number | GOREF name | Count |
| --- | --- | --- |
| GO_REF-0000108 | Automatic assignment of GO terms using logical inference, based on on inter-ontology links. | 1040 |
| GO_REF-0000003 | Gene Ontology annotation based on Enzyme Commission mapping | 1111 |
| GO_REF-0000002 | Gene Ontology annotation through association of InterPro records with GO terms. | 11453 |
| GO_REF-0000038 | OBSOLETE Gene Ontology annotation based on automatic assignment of UniProtKB keywords in UniProtKB/TrEMBL entries. | 7386 |
| GO_REF-0000104 | Electronic Gene Ontology annotations created by transferring manual GO annotations between related proteins based on shared sequence features. | 4719 |
| GO_REF-0000041 | Gene Ontology annotation based on UniPathway vocabulary mapping. | 358 |
| GO_REF-0000037 | OBSOLETE Gene Ontology annotation based on manual assignment of UniProtKB keywords in UniProtKB/Swiss-Prot entries. | 2873 |
| GO_REF-0000039 | OBSOLETE Gene Ontology annotation based on the manual assignment of UniProtKB Subcellular Location terms in UniProtKB/Swiss-Prot entries. | 347 |
| GO_REF-0000040 | OBSOLETE Gene Ontology annotation based on the automatic assignment of UniProtKB Subcellular Location terms in UniProtKB/TrEMBL entries. | 384 |
| GO_REF-0000024 | Manual transfer of experimentally-verified manual GO annotation data to orthologs by curator judgment of sequence similarity. | 8 |

A summary of the evidence used in Biocyc GO annotations. Count refers to the number of GO terms supported with each evidence code.

Table S2

| ECO number | ECO name | Count |
| --- | --- | --- |
| ECO:0000259 | match to InterPro member signature evidence used in automatic assertion | 10362 |
| ECO:0000136 | nucleic acid binding evidence | 1 |
| ECO:0000096 | electrophoretic mobility shift assay evidence | 1 |
| ECO:0000044 | sequence similarity evidence | 2 |
| ECO:0000250 | sequence similarity evidence used in manual assertion | 4 |
| ECO:0000016 | loss-of-function mutant phenotype evidence | 11 |
| ECO:0000315 | mutant phenotype evidence used in manual assertion | 13 |
| ECO:0000314 | direct assay evidence used in manual assertion | 2 |
| ECO:0000012 | functional complementation evidence | 4 |
| ECO:0000005 | enzymatic activity assay evidence | 3 |
| ECO_0000024 | protein binding evidence | 2 |

A summary of the evidence used in the Pseudomonas Genome Database GO annotations. Count refers to the number of GO terms supported with each evidence code.

Table S3

| ECO number | ECO name | Count |
| --- | --- | --- |
| ECO:0000305 | curator inference used in manual assertion | 346 |
| ECO:0000269 | experimental evidence used in manual assertion | 535 |
| ECO:0000303 | author statement without traceable support used in manual assertion | 66 |
| ECO:0000312 | imported information used in manual assertion | 51 |
| ECO:0007744 | combinatorial computational and experimental evidence used in manual assertion | 140 |
| ECO:0000255 | match to sequence model evidence used in manual assertion | 8378 |
| ECO:0007829 | combinatorial computational and experimental evidence used in automatic assertion | 1045 |
| ECO:0000353 | physical interaction evidence used in manual assertion | 41 |
| ECO:0000314 | direct assay evidence used in manual assertion | 55 |
| ECO:0000250 | sequence similarity evidence used in manual assertion | 483 |
| ECO:0000315 | mutant phenotype evidence used in manual assertion | 27 |
| ECO:0000256 | match to sequence model evidence used in automatic assertion | 31063 |
| ECO:0000313 | imported information used in automatic assertion | 39931 |
| ECO:0000259 | match to InterPro member signature evidence used in automatic assertion | 4218 |
| ECO:0008006 | deep learning neural network method evidence used in automatic assertion | 800 |
| ECO:0000270 | expression pattern evidence used in manual assertion | 1 |

A summary of the evidence used in Uniprot GO annotations. Count refers to the number of GO terms supported with each evidence code.

Table S4

| Guilt by Association Similarity Model |
| --- |
| STRING: coexpression transferred |
| STRING: combined score |
| STRING: cooccurence |
| STRING: database transferred |
| STRING: database |
| STRING: experiments transferred |
| STRING: fusion |
| STRING: homology |
| STRING: neighborhood transferred |
| STRING: neighborhood |
| STRING: textmining transferred |
| STRING: textmining |
| bitscore |
| proteomic coexpression |
| proteomic cooccurence |
| sequence feature similarity |
| TM-score |
| Robinson-Foulds tree similarity |
| number of shared taxa |
| shared operon |

The vector of scores used in the protein-protein similarity model for the guilt by association arm of the analysis.

Table S5

| Guilt by Association Term Transfer Model | |
| --- | --- |
| Similarity Measure | Summary Statistics |
| STRING: coexpression transferred | sum, max |
| STRING: combined score | sum, max |
| STRING: cooccurence | sum, max |
| STRING: database transferred | sum, max |
| STRING: database | sum, max |
| STRING: experiments transferred | sum, max |
| STRING: fusion | sum, max |
| STRING: homology | sum, max |
| STRING: neighborhood transferred | sum, max |
| STRING: neighborhood | sum, max |
| STRING: textmining transferred | sum |
| STRING: textmining | sum, max |
| bitscore | sum, max |
| proteomic coexpression | sum, min |
| proteomic cooccurence | sum |
| sequence feature similarity | sum, min |
| TM-score | sum, max |
| Robinson-Foulds tree similarity | sum, max |
| number of shared taxa | sum |
| shared operon | count |
| number of hits | - |

Summary statistics used as prediction features for the guilt by association annotation model.

Table S6

| Structural Similarity Term Transfer Model | |
| --- | --- |
| Similarity Measure | Summary Statistics |
| TM-score | sum, max |
| RMSD | min, mean |
| % sequence ID | sum, max |
| % non-gap | sum, max |
| is eukaryote | any, all |
| phylogenetic similarity | min, mean |
| number of hits | - |

Summary statistics used as prediction features for the structural similarity annotation model.

Table S7

| Protein | Hypothesized Function |
| --- | --- |
| PP_2483 | Nucleotidyl Transferase |
| PP_1372 | DNA Translocase |
| PP_4312 | Phosphotase |
| PP_4586 | M1 Peptidase |
| PP_27447 | Long Chain Fatty Acid CoA Ligase |
| PP_2666 | Phosphotase, Reductase, or Hydrolase |
| PP_2447 | Peptidase |
| PP_0512 | Pilotin |
| PP_0717 | Ferratin |
| PP_3777 | Methyltransferase |
| PP_2099 | Ribosomal Binding Protien |
| PP_2200 | DinB DNA Polymerase or Thiol S-Transferase |
| PP_3784 | Chorismatase |
| PP_3785 | WD-40 Fold Scaffold Protein |
| PP_3787 | Chalcone Synthase |
| PP_5628 | Acyl Carrier Protein |
| PP_3800 | Pilin |
| PP_4969 | Chitooligosaccharide Deacetylase |
| PP_0576 | Porin, possibly involved in biofilm formation |
| PP_2007 | P-47 like Lipid Binding Protein |
| PP_2363 | CsuE like Biofilm Forming Protein |
| PP_2853 | FapF like Biofilm Amyloid Exporter |
| PP_3350 | Porin |
| PP_3954 | Periplasmic Substrate Binding Protein |
| PP_5531 | DinB Superfamily Metalloenzyme |

Hypothesized functions based on a manual analysis of the data collected for both predictive models and visualizations of RUPEE hit structural alignments using the PDB pairwise structural alignment tool.
