# Supplementary material for: Multi-Omics integration can be used to rescue metabolic information for some of the dark region of the Pseudomonas putida proteome
Source: BMC Genomics. 2024 Mar 11;25:267. doi: 10.1186/s12864-024-10082-y (PMC10926591; doi:10.1186/s12864-024-10082-y)
Supplement: Supplementary file 4 — Additional file 4. This file contains the notes from the manual reanalysis of specific proteins in Pseudomonas putida. These proteins were chosen for reanalysis based on either the large number of predicted terms from the automatic models or based on requests from collaborators. [file 12864_2024_10082_MOESM4_ESM.docx]

**Additional File 4. This file contains the notes from the manual reanalysis of specific proteins in *Pseudomonas putida*. These proteins were chosen for reanalysis based on either the large number of predicted terms from the automatic models or based on requests from collaborators.**

**PP_2483**:

predicted terms from structural homology model:

GO:0003824 catalytic activity

GO:0005488 binding

GO:0009987 cellular process

GO:0008152 metabolic process

GO:0110165 cellular anatomical entity

GO:0044238 primary metabolic process

GO:0044281 small molecule metabolic process

GO:0097159 organic cyclic compound binding

GO:0016740 transferase activity

GO:0044237 cellular metabolic process

GO:0009058 biosynthetic process

GO:1901363 heterocyclic compound binding

GO:0071704 organic substance metabolic process

GO:0036094 small molecule binding

GO:0043167 ion binding

GO:0006807 nitrogen compound metabolic process

GO:0043168 anion binding

GO:1901576 organic substance biosynthetic process

GO:0006793 phosphorus metabolic process

GO:0016772 transferase activity, transferring phosphorus-containing groups

GO:0044249 cellular biosynthetic process

GO:1901265 nucleoside phosphate binding

GO:1901135 carbohydrate derivative metabolic process

GO:0034641 cellular nitrogen compound metabolic process

GO:0046483 heterocycle metabolic process

GO:0006725 cellular aromatic compound metabolic process

GO:1901360 organic cyclic compound metabolic process

GO:1901564 organonitrogen compound metabolic process

GO:0016779 nucleotidyltransferase activity

GO:0006796 phosphate-containing compound metabolic process

GO:0000166 nucleotide binding

GO:0019637 organophosphate metabolic process

GO:0019438 aromatic compound biosynthetic process

GO:0018130 heterocycle biosynthetic process

GO:1901362 organic cyclic compound biosynthetic process

GO:0044271 cellular nitrogen compound biosynthetic process

GO:0006139 nucleobase-containing compound metabolic process

GO:0035639 purine ribonucleoside triphosphate binding

GO:0090407 organophosphate biosynthetic process

GO:0055086 nucleobase-containing small molecule metabolic process

GO:0034654 nucleobase-containing compound biosynthetic process

GO:0017076 purine nucleotide binding

GO:0032555 purine ribonucleotide binding

GO:0006753 nucleoside phosphate metabolic process

GO:0009117 nucleotide metabolic process

GO:1901293 nucleoside phosphate biosynthetic process

GO:0009165 nucleotide biosynthetic process

top structure hits

TM-score: 0.87, D5MNX3 Moba relate protein nan Mycobacterium sp. (strain DSM 3803 / JC1)

TM-score: 0.87, O53706 NTP_transf_3 domain-containing protein MT0386 Mycobacterium tuberculosis (strain CDC 1551 / Oshkosh)

TM-score: 0.85, O26628 NTP_transf_3 domain-containing protein MTH_528 Methanothermobacter thermautotrophicus (strain ATCC 29096 / DSM 1053 / JCM 10044 / NBRC 100330 / Delta H) (Methanobacterium thermoautotrophicum)

TM-score: 0.84, P9WKG9 2-C-methyl-D-erythritol 4-phosphate cytidylyltransferase, EC 2.7.7.60 (4-diphosphocytidyl-2C-methyl-D-erythritol synthase) (MEP cytidylyltransferase, MCT) ispD Rv3582c MTCY06G11.29c Mycobacterium tuberculosis (strain ATCC 25618 / H37Rv)

TM-score: 0.84, A0R560 2-C-methyl-D-erythritol 4-phosphate cytidylyltransferase, EC 2.7.7.60 (4-diphosphocytidyl-2C-methyl-D-erythritol synthase) (MEP cytidylyltransferase, MCT) ispD MSMEG_6076 Mycolicibacterium smegmatis (strain ATCC 700084 / mc(2)155) (Mycobacterium smegmatis)

predicted terms from within species model:

GO:0008152 metabolic process

GO:0003824 catalytic activity

GO:0110165 cellular anatomical entity

GO:0005488 binding

GO:0016740 transferase activity

GO:0043167 ion binding

GO:0016772 transferase activity, transferring phosphorus-containing groups

GO:0043169 cation binding

GO:0046872 metal ion binding

top predicted similar proteins

PP_4230 8.69 NTP_transf_3 domain-containing protein

PP_0726 6.67 Transferase

PP_1792 6.1 Glycosyl transferase, group 2 family protein

PP_5411 6.1 Bifunctional protein GlmU [Includes: UDP-N-acetylglucosamine pyrophosphorylase (EC 2.7.7.23) (N-acetylglucosamine-1-phosphate uridyltransferase); Glucosamine-1-phosphate N-acetyltransferase (EC 2.3.1.157)]

interproscan results

"PP_2483","hypothetical protein","CDD","cd04182","GT_2_like_f","","12","193","3.37523E-63","","5.36-75.0",""

"PP_2483","hypothetical protein","Gene3D","G3DSA:3.90.550.10","","IPR029044","7","196","4.1E-48","Nucleotide-diphospho-sugar transferases","5.36-75.0",""

"PP_2483","hypothetical protein","SUPERFAMILY","SSF53448","","IPR029044","9","193","9.86E-26","Nucleotide-diphospho-sugar transferases","5.36-75.0",""

"PP_2483","hypothetical protein","Pfam","PF12804","MobA-like NTP transferase domain","IPR025877","12","171","3.2E-34","MobA-like NTP transferase","5.36-75.0",""

proteomic evidence for existence: False

observed as differentially expressed: False

Notes:

The structural homology hits here are high quality. The first hit, D5MNX3, is a tuberculosis mobA protein which converts molybdenum cofactor (MoCo) to *bis-*molybdopterin guanine dinucleotide cofactor. The second protein O53706 is a mocA homolog also in tuberculosis. mocA catalyzes the formation of molybdopterin cytosine dinucleotide cofactor from MoCo. These are clearly closely related enzymes both of which have the function of adding nucleotides to MoCo.

The third hit, O26628, is a nucleotidyl transferase from an archaeon *Methanothermobacter thermautotrophicus* str. Delta H. little other information could be found about this one.

The fourth and fifth hits are orthologs of each other from two *Mycobacterium* species *tuberculosis* and *smegmeatis*. They are 2-C-methyl-D-erythritol 4-phosphate cytidylyltransferases or ispD, which catalyze the third step in the MEP isopentenyl diphosphate (IPP) synthesis pathway which takes 2-C-methyl-d-erythritol 4-phosphate to 4-diphosphocytidyl-2-C-methyl-d-erythritol. This again involves the addition of a nucleotide to a small molecule. These two hits are of particular interest as IPP synthesis is a crucial upstream component of synthesizing various industrially relevant isoprenoids like carotenoids and sterols.

The two best functional similarity hits are PP_4230 and PP_0726 which are also annotated as being a mobA like nucleotidyl transferases but with next to no other information about them. Both of these proteins are reasonably structurally similar to PP_2483. Both of the next two functional similarity hits have a subset of the structure that looks somewhat similar and they are both nucleotidyl transferases of some flavor.

Together these data seem to indicate that this is a small soluble mobA like nucleotidyl transferase but I can’t narrow down the substrate much from there.

**PP_1372**:

predicted terms from structural homology model:

GO:0003824 catalytic activity

GO:0005488 binding

GO:0009987 cellular process

GO:0008152 metabolic process

GO:0110165 cellular anatomical entity

GO:0044238 primary metabolic process

GO:0044237 cellular metabolic process

GO:0071704 organic substance metabolic process

GO:0036094 small molecule binding

GO:0097159 organic cyclic compound binding

GO:1901363 heterocyclic compound binding

GO:0097367 carbohydrate derivative binding

GO:0006807 nitrogen compound metabolic process

GO:0043167 ion binding

GO:0043168 anion binding

GO:1901265 nucleoside phosphate binding

GO:0046483 heterocycle metabolic process

GO:1901360 organic cyclic compound metabolic process

GO:0006725 cellular aromatic compound metabolic process

GO:0034641 cellular nitrogen compound metabolic process

GO:0000166 nucleotide binding

GO:0035639 purine ribonucleoside triphosphate binding

GO:0006139 nucleobase-containing compound metabolic process

GO:0032553 ribonucleotide binding

GO:0017076 purine nucleotide binding

GO:0032555 purine ribonucleotide binding

GO:0030554 adenyl nucleotide binding

GO:0032559 adenyl ribonucleotide binding

top structure hits

TM-score: 0.63, Q04230 TrwB (TrwB protein) trwB Escherichia coli

TM-score: 0.63, B0KAW2 Type IV secretory pathway VirB4 components-like protein Teth39_0060 Thermoanaerobacter pseudethanolicus (strain ATCC 33223 / 39E) (Clostridium thermohydrosulfuricum)

TM-score: 0.62, Q97WG8 DNA double-strand break repair helicase HerA, EC 3.6.4.12 herA SSO2251 Saccharolobus solfataricus (strain ATCC 35092 / DSM 1617 / JCM 11322 / P2) (Sulfolobus solfataricus)

TM-score: 0.6, A0A210BW77 DNA phosphorothioation-dependent restriction protein DptH dptH A6592_17195 Escherichia coli

TM-score: 0.44, Q9I0M3 DNA translocase FtsK ftsK PA2615 Pseudomonas aeruginosa (strain ATCC 15692 / DSM 22644 / CIP 104116 / JCM 14847 / LMG 12228 / 1C / PRS 101 / PAO1)

interproscan results

"PP_1372","hypothetical protein","Pfam","PF05872","Bacterial protein of unknown function (DUF853)","IPR033186","11","491","0.0","Helicase HerA-like C-terminal","5.36-75.0",""

"PP_1372","hypothetical protein","Gene3D","G3DSA:3.40.50.300","","","206","385","1.7E-18","","5.36-75.0",""

"PP_1372","hypothetical protein","Gene3D","G3DSA:3.40.50.300","","","2","95","3.1E-8","","5.36-75.0",""

"PP_1372","hypothetical protein","SMART","SM00382","","IPR003593","25","331","0.0096","AAA+ ATPase domain","5.36-75.0",""

"PP_1372","hypothetical protein","MobiDBLite","mobidb-lite","consensus disorder prediction","","424","445","-","","5.36-75.0",""

"PP_1372","hypothetical protein","SUPERFAMILY","SSF52540","","IPR027417","6","387","6.72E-49","P-loop containing nucleoside triphosphate hydrolase","5.36-75.0",""

proteomic evidence for existence: True

observed as differentially expressed: True

Q04230 is a trwB bacterial conjugation protein from *E. coli* which hexamerizes to form a putative transmembrane pore that passes DNA across membranes. This complex is believed to have helicase activity and unwinds DNA before passing it through the pore, this mechanism requires the capacity to hydrolyze ATP. The complex structure is similar to both ring helicases and F­_1_-ATPases. The middling TM-score is somewhat misleading as the solved structure is missing the transmembrane helix It appears that the sequence for that helical section is present in the alphafold prediction of PP_1372 however it ended up being laid alongside what would have been the solvent accessible surface of the hexamer. This is likely an artifact of the relative lack of transmembrane structures in alphafold’s training set.

B0KAW2 is a VirB4 ATPase and a component of the type IV secretion system in *Thermoanaerobacter pseudethanolicus*. VirB4 sits inside on the cytoplasmic side of the double membrane spanning pore and acts as a molecular motor to drive the handoff of DNA between two other carrier proteins. This protein aligns well with Q04230 but naturally lacks the transmembrane helix that was excluded from crystallization there, which seems to be present in PP_1372. It is worth noting that hexameric forms of VirB4s have been reported in the literature.

Q97WG8 is a hexameric ATPase from the archaeon *Saccharolobus solfataricus* P2 that is involved as a ring DNA translocase in double strand break repair. It’s got a small beta barrel and helix structure in the spot where trwB’s transmembrane domain and PP_1372’s nonsense loop are (fairly n-terminal), which means that inferring a transmembrane helix for PP_1372 is not necessarily a good idea. The barrel-and-helix (aka HAS-barrel) domain provides an interface for other proteins in the repair complex. The central pore in this hexamer is much larger than that observed in trwB, as it can pass B form dsDNA while trwB can only pass ssDNA, so it may be informative to attempt running the hexamer through alphafold to see if it ends up looking more like one or the other.

A0A210BW77 is a DNA phosphorothioation-dependent restriction protein DptH from *E. coli*. It appears to have the same basic folds as all of the previous proteins, but this is only the c-terminal 375aa of the full length 1687aa protein. Which makes me somewhat skeptical that I can make direct functional inferences from this hit. Additionally, there’s not an associated paper and I’m having trouble finding more information about this gene.

The alignment quality drops off hard after that, so I don’t think that I’ll get any more informed by analyzing these further. I’m reasonably willing to bet that this is a translocase of some flavor, but this selection of proteins doesn’t narrow down what processes it may be involved in all that much and the within species model isn’t giving me any clues. Trying the hexamer fold with alphafold will likely take quite a while but I suppose it’s worth the attempt. It turns out it wasn’t alphafold choked on that amount of data.

**PP_4312**:

predicted terms from structural homology model:

GO:0003824 catalytic activity

GO:0009987 cellular process

GO:0005488 binding

GO:0008152 metabolic process

GO:0110165 cellular anatomical entity

GO:0044238 primary metabolic process

GO:0044237 cellular metabolic process

GO:0071704 organic substance metabolic process

GO:0016787 hydrolase activity

GO:0140096 catalytic activity, acting on a protein

GO:0006807 nitrogen compound metabolic process

GO:0044281 small molecule metabolic process

GO:0009058 biosynthetic process

GO:0043170 macromolecule metabolic process

GO:0006793 phosphorus metabolic process

GO:1901564 organonitrogen compound metabolic process

GO:0016788 hydrolase activity, acting on ester bonds

GO:1901576 organic substance biosynthetic process

GO:0046483 heterocycle metabolic process

GO:0044249 cellular biosynthetic process

GO:0006725 cellular aromatic compound metabolic process

GO:0034641 cellular nitrogen compound metabolic process

GO:0043412 macromolecule modification

GO:0006796 phosphate-containing compound metabolic process

GO:0042578 phosphoric ester hydrolase activity

GO:0019538 protein metabolic process

GO:0016791 phosphatase activity

top structure hits

TM-score: 0.71, P9WIC7 Glucosyl-3-phosphoglycerate phosphatase, EC 3.1.3.85 (Mannosyl-3-phosphoglycerate phosphatase, EC 3.1.3.70) gpgP Rv2419c Mycobacterium tuberculosis (strain ATCC 25618 / H37Rv)

TM-score: 0.7, Q96HS1 Serine/threonine-protein phosphatase PGAM5, mitochondrial, EC 3.1.3.16 (Bcl-XL-binding protein v68) (Phosphoglycerate mutase family member 5) PGAM5 Homo sapiens (Human)

TM-score: 0.66, Q9ALU0 Phosphoglycerate mutase yhfR Geobacillus stearothermophilus (Bacillus stearothermophilus)

TM-score: 0.65, Q7ZVE3 Fructose-2,6-bisphosphatase TIGAR B, EC 3.1.3.46 (TP53-induced glycolysis and apoptosis regulator B) tigarb si:ch211-240j22.3 zgc:56074 Danio rerio (Zebrafish) (Brachydanio rerio)

TM-score: 0.65, P76502 Phosphohistidine phosphatase SixA, EC 3.1.3.- (RX6) sixA yfcW b2340 JW2337 Escherichia coli (strain K12)

interproscan results

"PP_4312","hypothetical protein","CDD","cd07040","HP","","43","176","0.00416106","","5.36-75.0",""

PP_4312 SignalP-5.0 signal_peptide 1 35 0.593707 . . Note=TAT

proteomic evidence for existence: False

observed as differentially expressed: False

P9WIC7 A Glucosyl-3-phosphoglycerate phosphatase from *M. tuberculosis* is involved in the biosynthesis of mycobacterial methylglucose lipopolysaccharides (MGLPs). Much of the small protein overlaps well with PP_4312 however there is a loop of approximately 36aa containing two small helices that is not found in the PUF.

The second best hit, Q96HS1, is a mitochondrial Serine/threonine-protein phosphatase in humans. It also has extra stuff in broadly the same spot, in this case the loop is not included in the crystal structure I believe that this is a membrane anchor, which may be what it is in P9WIC7 as well. in vivo the protein gets its membrane anchor cleaved and in the mito lumen dodecamerizes into rings which further grow into filaments much like microtubules. This filamentous behavior is strongly associated with mitochondrial morphology regulation, I doubt that this has much of an analog in *P. putida* where the internal morphology of the cell is remarkably different from mitochondria. That said the oligomerization of PP_4312 is quite likely as both Q96HS1 and P9WIC7 oligomerize.

The third best hit, Q9ALU0, is a phosphoglycerate mutase from *Geobacillus stearothermophilus*. That has very good structural similarity to P9WIC7. Its structure also has a paper associated with it which tells me that the loop present in these two proteins that is missing in PP_4312 is integral to the active site and contains at least one possibly two residues that do not seem to be present in the PUF. This is another phosphatase that apparently dephosphorylates large hydrophobic substrates. The paper goes on to say that substrate specificity is quite difficult to determine computationally in this superfamily as specificity tends to hinge on small differences in the active site.

Fourth best is a fructose-2,6-bisphosphatase from zebrafish, P76502. It is structurally extremely similar to Q9ALU0 and P9WIC7 and is another metabolite phosphatase.

The fifth best hit is to P76502 and unlike any of the previous proteins the catalytic group seems to match better, and the lower TM-score is due to somewhat higher structural slop throughout the alignment. The better match in the catalytic domain is mostly due to size however as the location of the loop in P76502 is occupied by an extended, but adjacent loop in the PUF. It is a protein histidine phosphatase SixA from *E. coli*. Of interest here is that this is involved in a His-Asp phosporelay in ArcB-ArcA signaling under anaerobic growth. To me this looks like a convergent evolution of active site architecture which may indicate that this is a protein phosphatase.

**PP_5421**:

predicted terms from within species model:

GO:0044281 small molecule metabolic process

GO:0009056 catabolic process

GO:0006082 organic acid metabolic process

GO:0044282 small molecule catabolic process

GO:1901575 organic substance catabolic process

GO:0044248 cellular catabolic process

GO:1901564 organonitrogen compound metabolic process

GO:0016054 organic acid catabolic process

GO:0043436 oxoacid metabolic process

GO:1901565 organonitrogen compound catabolic process

GO:0019752 carboxylic acid metabolic process

GO:0016748 succinyltransferase activity

GO:0046395 carboxylic acid catabolic process

GO:0016749 N-succinyltransferase activity

GO:0006520 cellular amino acid metabolic process

GO:0043648 dicarboxylic acid metabolic process

GO:0006105 succinate metabolic process

GO:1901605 alpha-amino acid metabolic process

GO:0009063 cellular amino acid catabolic process

GO:0009064 glutamine family amino acid metabolic process

GO:1901606 alpha-amino acid catabolic process

GO:0009065 glutamine family amino acid catabolic process

GO:0006525 arginine metabolic process

GO:0006527 arginine catabolic process

top predicted similar proteins

PP_4333 7.09 CheW domain protein

PP_4946 6.15 Sodium/proline symporter (Proline permease)

PP_2045 6.04 Metallo-beta-lactamase family protein

proteomic evidence for existence: False

observed as differentially expressed: False

PP_4333 is involved in flagellum biosynthesis.

None of these proteins seem to have all that much to do with each other and only the CheW domain protein is a half decent hit.

**PP_4586**:

predicted terms from structural homology model:

GO:0003824 catalytic activity

GO:0009987 cellular process

GO:0005488 binding

GO:0008152 metabolic process

GO:0110165 cellular anatomical entity

GO:0044238 primary metabolic process

GO:0044237 cellular metabolic process

GO:0071704 organic substance metabolic process

GO:0097159 organic cyclic compound binding

GO:1901363 heterocyclic compound binding

GO:0097367 carbohydrate derivative binding

GO:0043167 ion binding

GO:0006807 nitrogen compound metabolic process

GO:0016787 hydrolase activity

GO:1901564 organonitrogen compound metabolic process

GO:0043170 macromolecule metabolic process

top structure hits

TM-score: 0.71, Q9RVZ5 Aminopeptidase N, EC 3.4.11.2 DR_0875 Deinococcus radiodurans (strain ATCC 13939 / DSM 20539 / JCM 16871 / LMG 4051 / NBRC 15346 / NCIMB 9279 / R1 / VKM B-1422)

TM-score: 0.7, Q7WVY1 Aminopeptidase N, EC 3.4.11.2 CPS_3470 Colwellia psychrerythraea (strain 34H / ATCC BAA-681) (Vibrio psychroerythus)

TM-score: 0.7, P09960 Leukotriene A-4 hydrolase, LTA-4 hydrolase, EC 3.3.2.6 (Leukotriene A(4) hydrolase) (Tripeptide aminopeptidase LTA4H, EC 3.4.11.4) LTA4H LTA4 Homo sapiens (Human)

TM-score: 0.5, A0A0H2YN38 F5/8 type C domain protein CPF_1489 Clostridium perfringens (strain ATCC 13124 / DSM 756 / JCM 1290 / NCIMB 6125 / NCTC 8237 / Type A)

TM-score: 0.5, A0A0F7RB18 Conserved domain protein (Wall-Associated protein) BA_5305 BASH2_00675 Bacillus anthracis

interproscan results

PP_4586 SignalP-5.0 signal_peptide 1 19 0.997462 . . .

proteomic evidence for existence: True

observed as differentially expressed: True

Notes:

Q9RVZ5 is an M1 aminopeptidase from *Deinococcus radiodurans* with a substrate preference for large hydrophobic residues, in particular tyrosine. There’s reasonable alignment between it and PP_4586 however the N-terminal β sandwich domain is twisted relative to each other, this may be an artifact from alphafold as the protein is made up of two domains that come together to form a deep cleft with the active site and alphafold tends not to do as good of a job positioning domains relative to each other. The strict sequence homology is not high but amino acid properties are maintained quite well and it is likely that this protein has a reasonably similar substrate spectrum to PP_4586. The substrate preference for aromatic, hydrophobic residues is intriguing in the context of lignin metabolism.

Q7WVY1 is also an M1 aminopeptidase, this time from *Colwellia psychrerythraea*, which is strongly cold adapted. The fit is better here but Q7WVY1 has a large α-helix rich c-terminal domain that is absent in PP_4586. Consistent with the cold adaptations of *C. psychrerythraea,* the loops on Q7WVY1 are consistently larger than those on PP_4586.

P09960 is a bifunctional hydrolase/aminopeptidase with overlapping active sites from humans. It has a similar C-terminal domain to Q7WVY1 and the fit for the rest of the protein is worse but still clearly in the same family. The fact that non-peptide hydrolase functionality is capable of evolving in this general fold structure is also intriguing in the context of lignin catabolism.

The latter two hits are not particularly good.


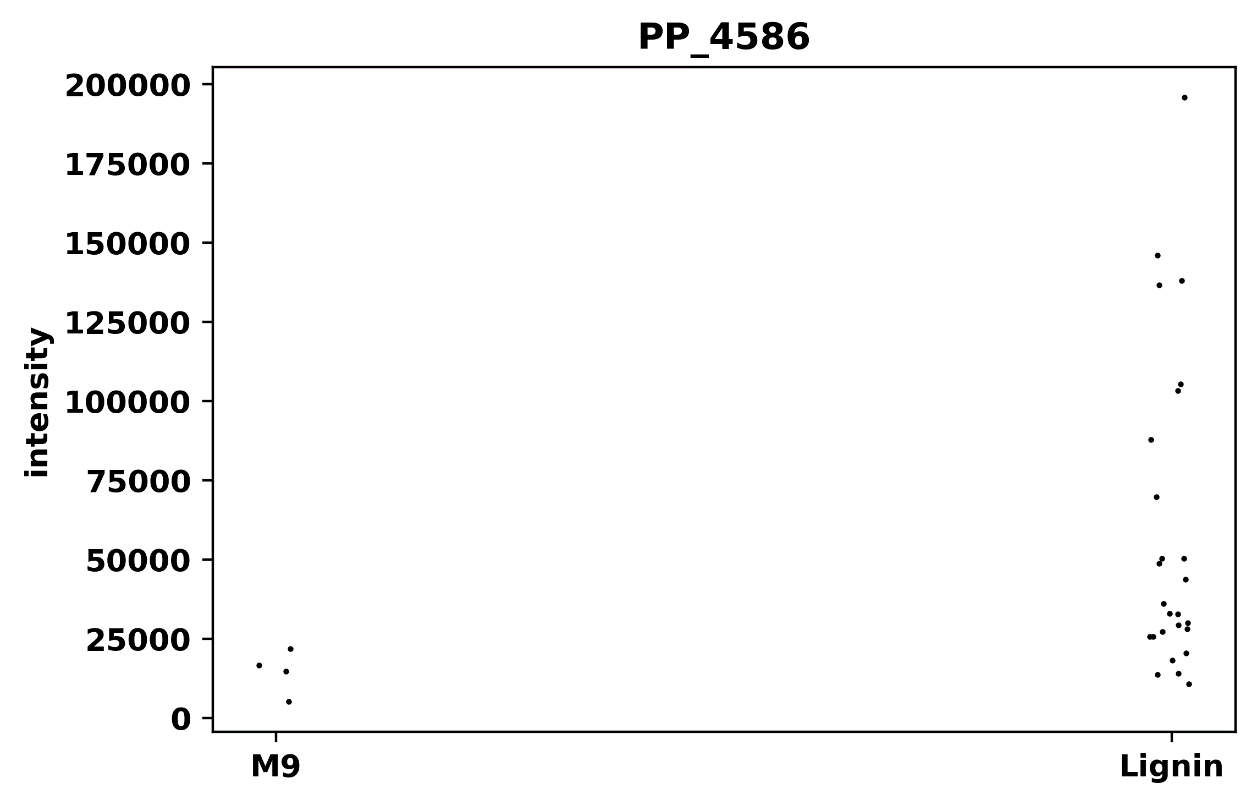

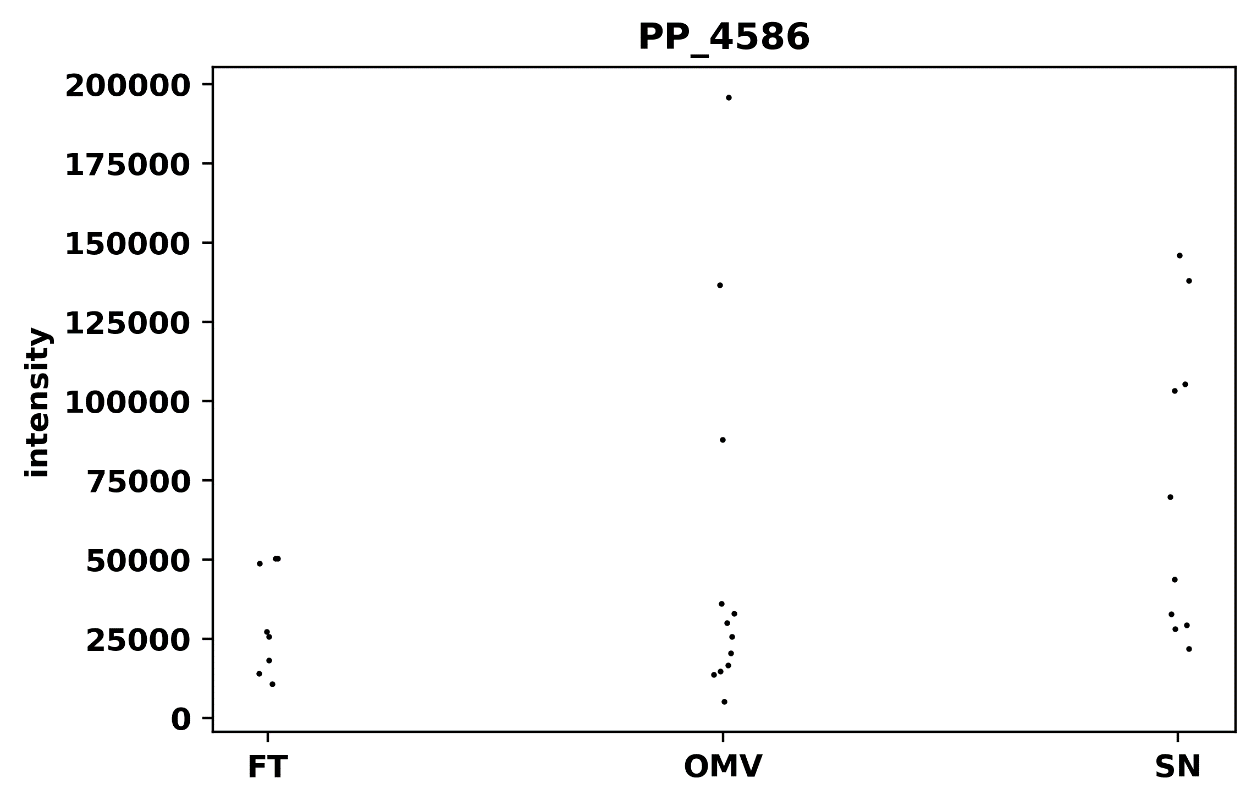
This is clearly a two domain M1 peptidase that likely works quite similarly to M1dr. Consistent with its signal peptide it is detected in the secretome. Interestingly its also detected and differentially expressed in OMVs.

It is out there but possible that PP_4586 acts directly on lignin. M1dr already has an unusually open active site which could allow it to work on full sized proteins or other large polymers and PP_4586 is predicted to have an even more open conformation, although that is of questionable reliability. Furthermore, the substrate preference of M1dr toward tyrosine and other bulky hydrophobic residues is not that far off from monolignols which are synthesized using phenylalanine as a starting material. It would be unlikely that it’s breaking C-C bonds but there are also plenty of C-O bonds in lignin that need hydrolyzing, and this could be a player there. A less dramatic interpretation is that its actually a peptidase that’s involved in breaking down the proteinaceous components of woody material.

The predicted terms are pleasingly consonant with the more detailed function prediction.

**PP_5484**

predicted terms from structural homology model:

GO:0003824 catalytic activity

GO:0009987 cellular process

GO:0008152 metabolic process

GO:0110165 cellular anatomical entity

GO:0016740 transferase activity

GO:0044237 cellular metabolic process

GO:0071704 organic substance metabolic process

GO:0006807 nitrogen compound metabolic process

GO:0034641 cellular nitrogen compound metabolic process

GO:0016765 transferase activity, transferring alkyl or aryl (other than methyl) groups

GO:1901564 organonitrogen compound metabolic process

GO:0006790 sulfur compound metabolic process

GO:0006575 cellular modified amino acid metabolic process

GO:0043603 cellular amide metabolic process

GO:0006518 peptide metabolic process

top structure hits

TM-score: 0.34, P36136 Sedoheptulose 1,7-bisphosphatase, EC 3.1.3.37 SHB17 YKR043C Saccharomyces cerevisiae (strain ATCC 204508 / S288c) (Baker's yeast)

TM-score: 0.34, A0A0J7JFD7 Acetoin utilization deacetylase AcuC Msub_13096 Marinobacter subterrani

TM-score: 0.34, O18598 Glutathione S-transferase, EC 2.5.1.18 (GST class-sigma) (Major allergen Bla g 5) (allergen Bla g 5) nan Blattella germanica (German cockroach) (Blatta germanica)

TM-score: 0.33, Q8NR03 Predicted glutathione S-transferase Cgl1264 Corynebacterium glutamicum (strain ATCC 13032 / DSM 20300 / BCRC 11384 / JCM 1318 / LMG 3730 / NCIMB 10025)

TM-score: 0.33, Q06A71 Glutathione transferase, EC 2.5.1.18 nan Fasciola hepatica (Liver fluke)

proteomic evidence for existence: True

observed as differentially expressed: False

Notes:

These hits are complete nonsense.

**PP_2747**

predicted terms from structural homology model:

GO:0003824 catalytic activity

GO:0005488 binding

GO:0009987 cellular process

GO:0008152 metabolic process

GO:0044238 primary metabolic process

GO:0097159 organic cyclic compound binding

GO:0044237 cellular metabolic process

GO:1901363 heterocyclic compound binding

GO:0071704 organic substance metabolic process

GO:0006807 nitrogen compound metabolic process

GO:0036094 small molecule binding

GO:0043167 ion binding

GO:0043168 anion binding

GO:1901564 organonitrogen compound metabolic process

GO:0000166 nucleotide binding

top structure hits

TM-score: 0.68, Q6NAE3 Putative long-chain-fatty-acid CoA ligase, EC 6.2.1.3 fadD1 RPA1242 Rhodopseudomonas palustris (strain ATCC BAA-98 / CGA009)

TM-score: 0.42, Q06210 Glutamine--fructose-6-phosphate aminotransferase [isomerizing] 1, EC 2.6.1.16 (D-fructose-6-phosphate amidotransferase 1) (Glutamine:fructose-6-phosphate amidotransferase 1, GFAT 1, GFAT1) (Hexosephosphate aminotransferase 1) GFPT1 GFAT GFPT Homo sapiens (Human)

TM-score: 0.42, Q9KJU4 Histidinol-phosphate aminotransferase, EC 2.6.1.9 (Imidazole acetol-phosphate transaminase) hisC Cgl2101 cg2304 Corynebacterium glutamicum (strain ATCC 13032 / DSM 20300 / BCRC 11384 / JCM 1318 / LMG 3730 / NCIMB 10025)

TM-score: 0.42, P9WML5 Putative phenylalanine aminotransferase, EC 2.6.1.- pat hisC2 Rv3772 MTCY13D12.06 Mycobacterium tuberculosis (strain ATCC 25618 / H37Rv)

TM-score: 0.42, P97084 Threonine-phosphate decarboxylase, EC 4.1.1.81 (L-threonine-O-3-phosphate decarboxylase) cobD STM0644 Salmonella typhimurium (strain LT2 / SGSC1412 / ATCC 700720)

interproscan results

"PP_2747","hypothetical protein","Gene3D","G3DSA:3.40.50.12780","","IPR042099","10","191","1.4E-11","AMP-dependent synthetase-like superfamily","5.36-75.0",""

"PP_2747","hypothetical protein","SUPERFAMILY","SSF56801","","","8","263","6.54E-15","","5.36-75.0",""

"PP_2747","hypothetical protein","Pfam","PF00501","AMP-binding enzyme","IPR000873","22","127","3.5E-9","AMP-dependent synthetase/ligase","5.36-75.0",""

"PP_2747","hypothetical protein","CDD","cd17641","LC_FACS_bac1","","35","294","4.18231E-9","","5.36-75.0",""

proteomic evidence for existence: False

observed as differentially expressed: False

Notes:

Only the first hit is reasonable. I’s a FadD1 Putative long-chain-fatty-acid CoA ligase which is involved in siderophore production and uptake. This functionality is backed up by interpro results, specifically IPR042099.

**PP_2666**:

predicted terms from structural homology model:

GO:0003824 catalytic activity

GO:0009987 cellular process

GO:0005488 binding

GO:0008152 metabolic process

GO:0110165 cellular anatomical entity

GO:0044238 primary metabolic process

GO:0044237 cellular metabolic process

GO:0071704 organic substance metabolic process

GO:0016787 hydrolase activity

GO:0006807 nitrogen compound metabolic process

GO:0097159 organic cyclic compound binding

GO:1901363 heterocyclic compound binding

GO:0043170 macromolecule metabolic process

GO:1901564 organonitrogen compound metabolic process

GO:0016788 hydrolase activity, acting on ester bonds

top structure hits

TM-score: 0.6, Q9M158 Rhodanese-like domain-containing protein 4, chloroplastic (Protein THYLAKOID RHODANESE-LIKE) (Sulfurtransferase 4, AtStr4) STR4 TROL At4g01050 F2N1.31 Arabidopsis thaliana (Mouse-ear cress)

TM-score: 0.6, Q6N3S9 Rhodanese domain-containing protein RPA3614 Rhodopseudomonas palustris (strain ATCC BAA-98 / CGA009)

TM-score: 0.6, P30305 M-phase inducer phosphatase 2, EC 3.1.3.48 (Dual specificity phosphatase Cdc25B) CDC25B CDC25HU2 Homo sapiens (Human)

TM-score: 0.59, Q9BY84 Dual specificity protein phosphatase 16, EC 3.1.3.16, EC 3.1.3.48 (Mitogen-activated protein kinase phosphatase 7, MAP kinase phosphatase 7, MKP-7) DUSP16 KIAA1700 MKP7 Homo sapiens (Human)

TM-score: 0.59, Q6Q1Q5 Sb(V)-As(V) reductase ACR2 Leishmania major

interproscan results

"PP_2666","hypothetical protein","CDD","cd00158","RHOD","","61","171","7.19817E-11","","5.36-75.0",""

"PP_2666","hypothetical protein","SUPERFAMILY","SSF52821","","IPR036873","54","186","1.83E-20","Rhodanese-like domain superfamily","5.36-75.0",""

"PP_2666","hypothetical protein","Gene3D","G3DSA:3.40.250.10","","IPR036873","39","179","9.2E-17","Rhodanese-like domain superfamily","5.36-75.0",""

"PP_2666","hypothetical protein","TIGRFAM","TIGR03865","PQQ_CXXCW: PQQ-dependent catabolism-associated CXXCW motif protein","IPR022376","22","182","2.4E-71","PQQ-dependent catabolism-associated CXXCW motif","5.36-75.0",""

"PP_2666","hypothetical protein","ProSiteProfiles","PS50206","Rhodanese domain profile.","IPR001763","68","179","11.96","Rhodanese-like domain","5.36-75.0",""

"PP_2666","hypothetical protein","Pfam","PF00581","Rhodanese-like domain","IPR001763","63","172","4.3E-7","Rhodanese-like domain","5.36-75.0",""

PP_2666 SignalP-5.0 signal_peptide 1 27 0.996296 . . .

proteomic evidence for existence: True

observed as differentially expressed: True


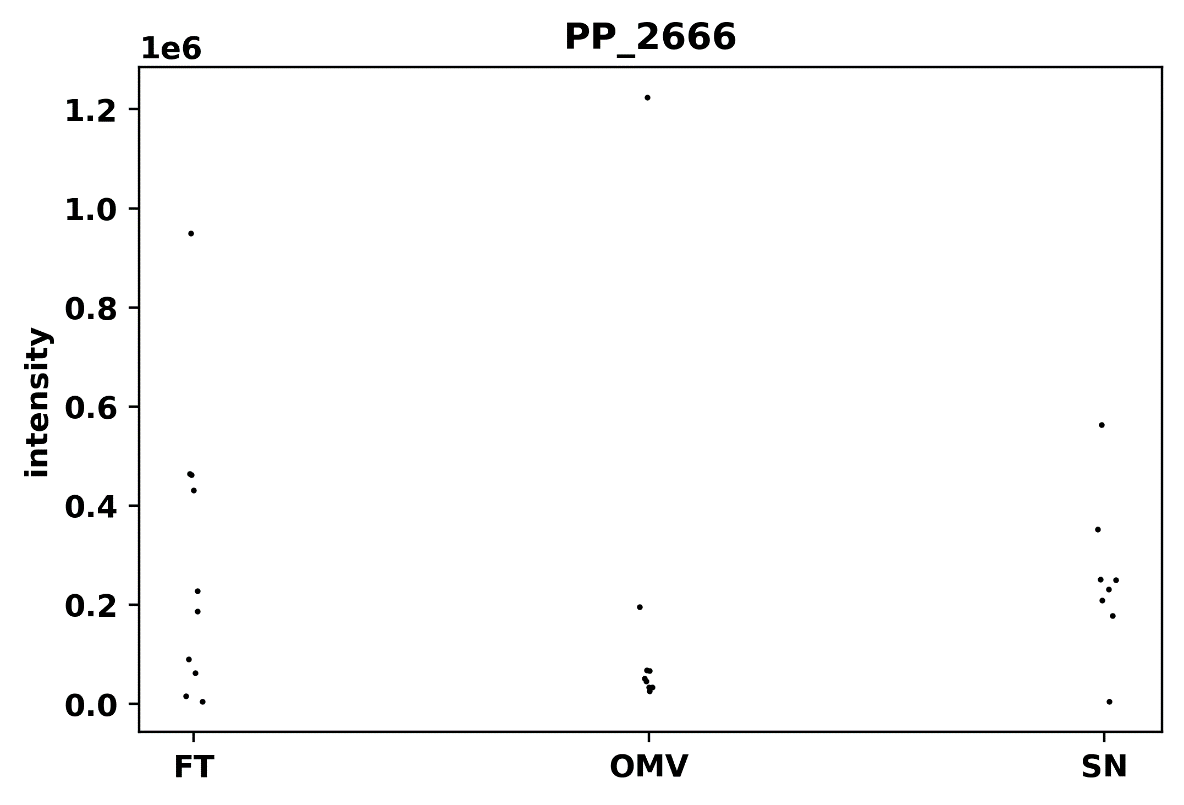


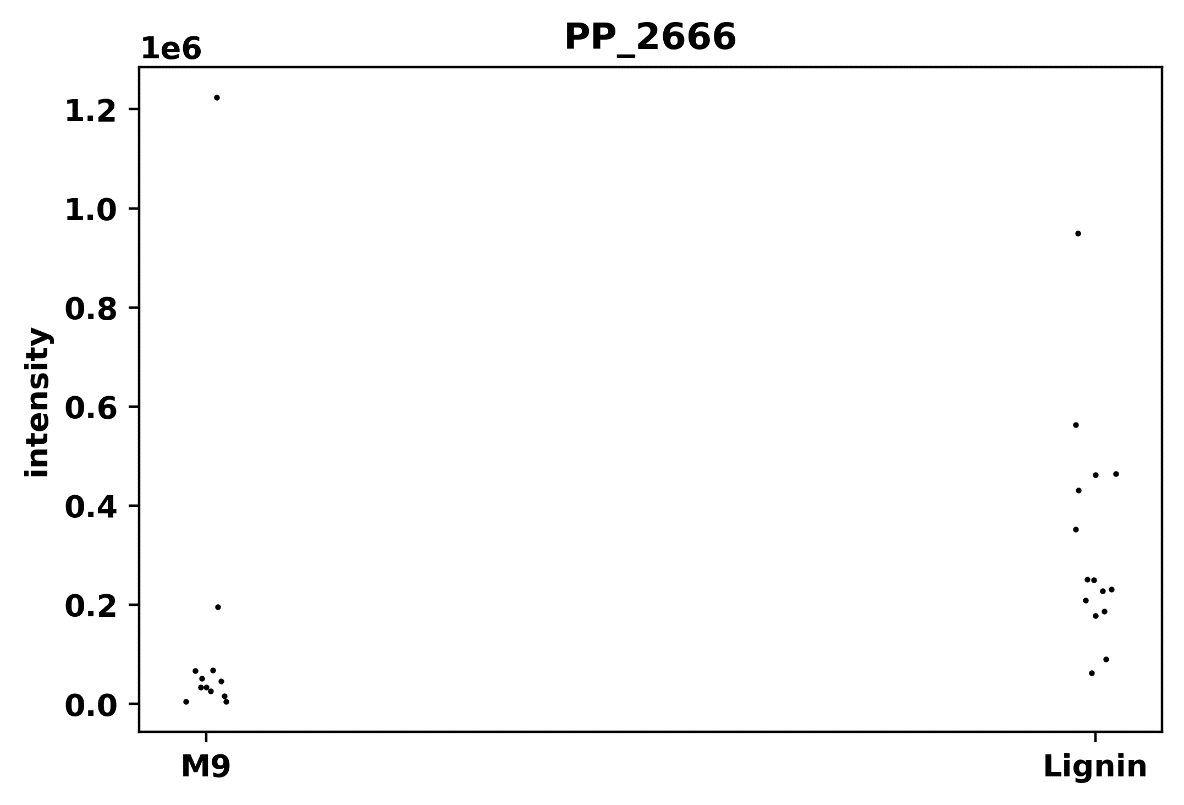


Notes:

This has the structure of a single rhodanese domain which means that it could be a rhodanese but is more likely a protein phosphatase or hydrolase. In this case it is most likely a phosphatase as most of the hits are to phosphatases. That said it also hits fairly well against As/Sb Reductase LmACR2 from *Leishmania major*. This protein also has phosphatase activity, raising the possibility of bifunctionality. Consistent with the presence of a signal peptide the protein is in fact excreted and interestingly it seems to be upregulated in response to lignin. I do not think that it can be reasonably inferred to be present in OMVs as the single high value is not corroborated by replicates. The CXXCW motif identified by interproscan indicates that this protein is responsive to redox conditions. It seems somewhat unlikely that there is significant need for phosphoregulation of secreted proteins. That could be evidence for a function other than protein phosphatase, such as the As/Sb Reductase activity that one of its structural homologs exhibits.

Side Note:

All proteomic intensity values have come from PXD016028 and I’ve not normalized them in any way. This had me worried that the number of PUFs seemingly upregulated in lignin media was simply due to a systematically higher. As seen below, this is not the case:


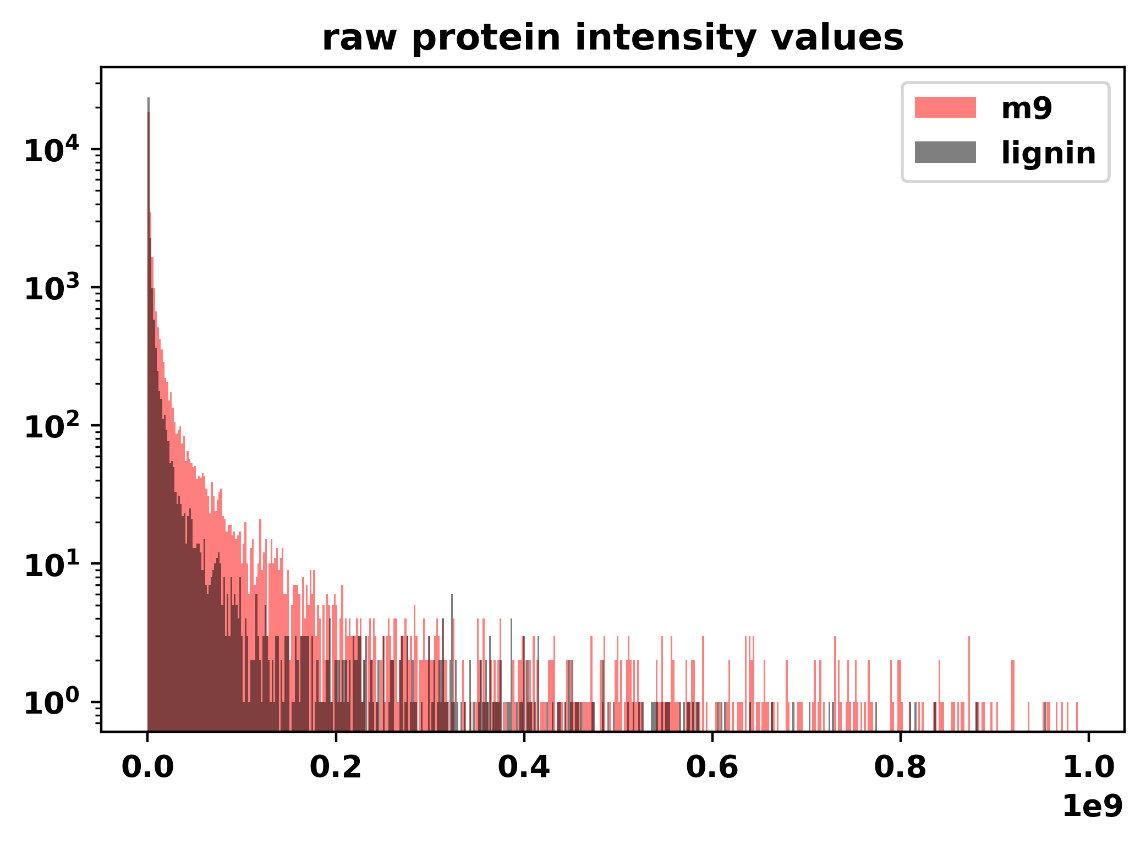


This raises the question of whether or not PUFs are overrepresented in lignin rich media.


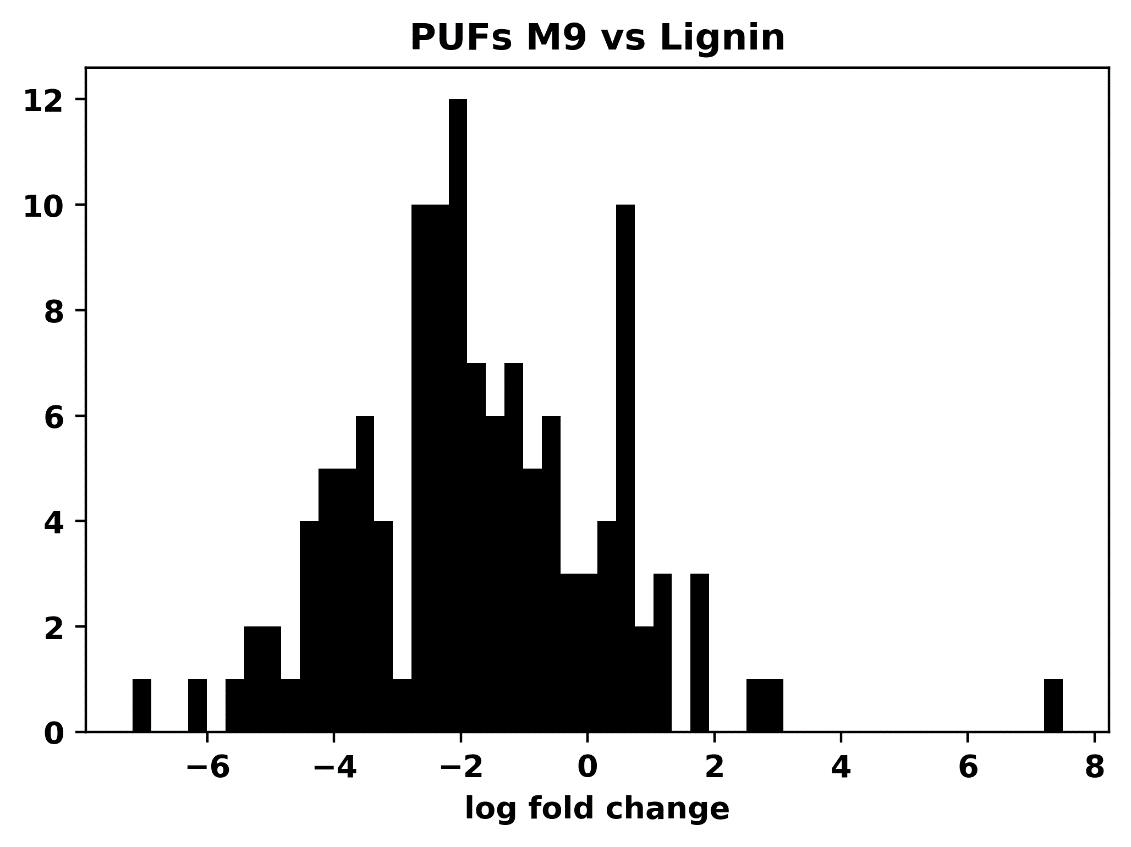


No, probably not. But let’s see if a centered log ratio transform changes things:


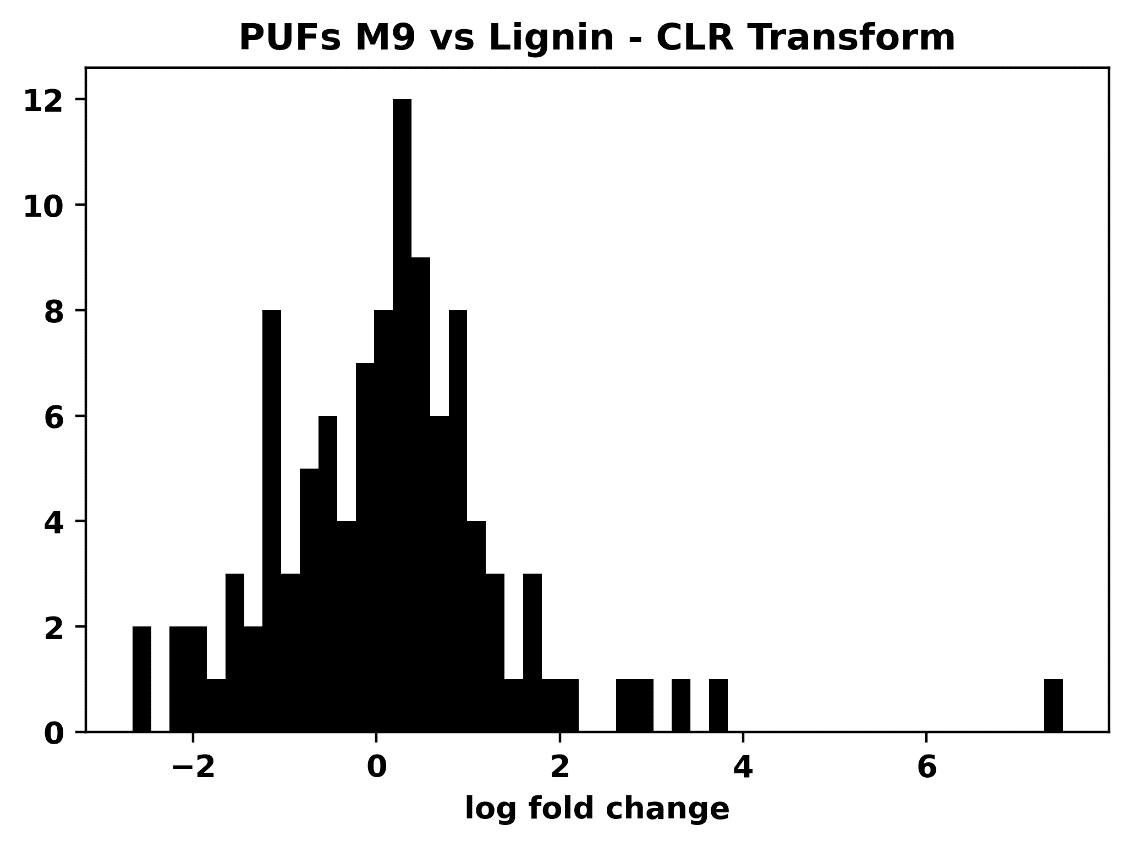


Also, no.

**PP_2447**:

predicted terms from structural homology model:

GO:0003824 catalytic activity

GO:0009987 cellular process

GO:0005488 binding

GO:0008152 metabolic process

GO:0110165 cellular anatomical entity

GO:0044238 primary metabolic process

GO:0044237 cellular metabolic process

GO:0071704 organic substance metabolic process

GO:0097159 organic cyclic compound binding

GO:1901363 heterocyclic compound binding

GO:0043167 ion binding

GO:0016740 transferase activity

GO:0006807 nitrogen compound metabolic process

GO:1901564 organonitrogen compound metabolic process

GO:0034641 cellular nitrogen compound metabolic process

top structure hits

TM-score: 0.68, Q8YY76 Glutathione gamma-glutamylcysteinyltransferase, EC 2.3.2.15 alr0975 alr0975 Nostoc sp. (strain PCC 7120 / SAG 25.82 / UTEX 2576)

TM-score: 0.62, A3DCU1 ABC-type bacteriocin transporter Cthe_0534 Acetivibrio thermocellus (strain ATCC 27405 / DSM 1237 / JCM 9322 / NBRC 103400 / NCIMB 10682 / NRRL B-4536 / VPI 7372) (Clostridium thermocellum)

TM-score: 0.61, P81297 Staphopain A, EC 3.4.22.48 (Staphylococcal cysteine proteinase A) (Staphylopain A) sspP scpA Staphylococcus aureus

TM-score: 0.61, P0C1S6 Staphopain B, EC 3.4.22.- (Staphylococcal cysteine proteinase B) (Staphylopain B) sspB Staphylococcus aureus

TM-score: 0.58, A0A2U7NR52 AvrRpt2 nan Erwinia amylovora (Fire blight bacteria)

interproscan results

"PP_2447","hypothetical protein","Gene3D","G3DSA:3.90.70.10","","","71","220","2.8E-6","","5.36-75.0",""

"PP_2447","hypothetical protein","CDD","cd02549","Peptidase_C39A","IPR039563","69","205","1.51154E-32","Peptidase C39 single domain protein","5.36-75.0",""

"PP_2447","hypothetical protein","Coils","Coil","","","228","244","-","","5.36-75.0",""

PP_2447 SignalP-5.0 lipoprotein_signal_peptide 1 44 0.912614 . . .

proteomic evidence for existence: True

observed as differentially expressed: True


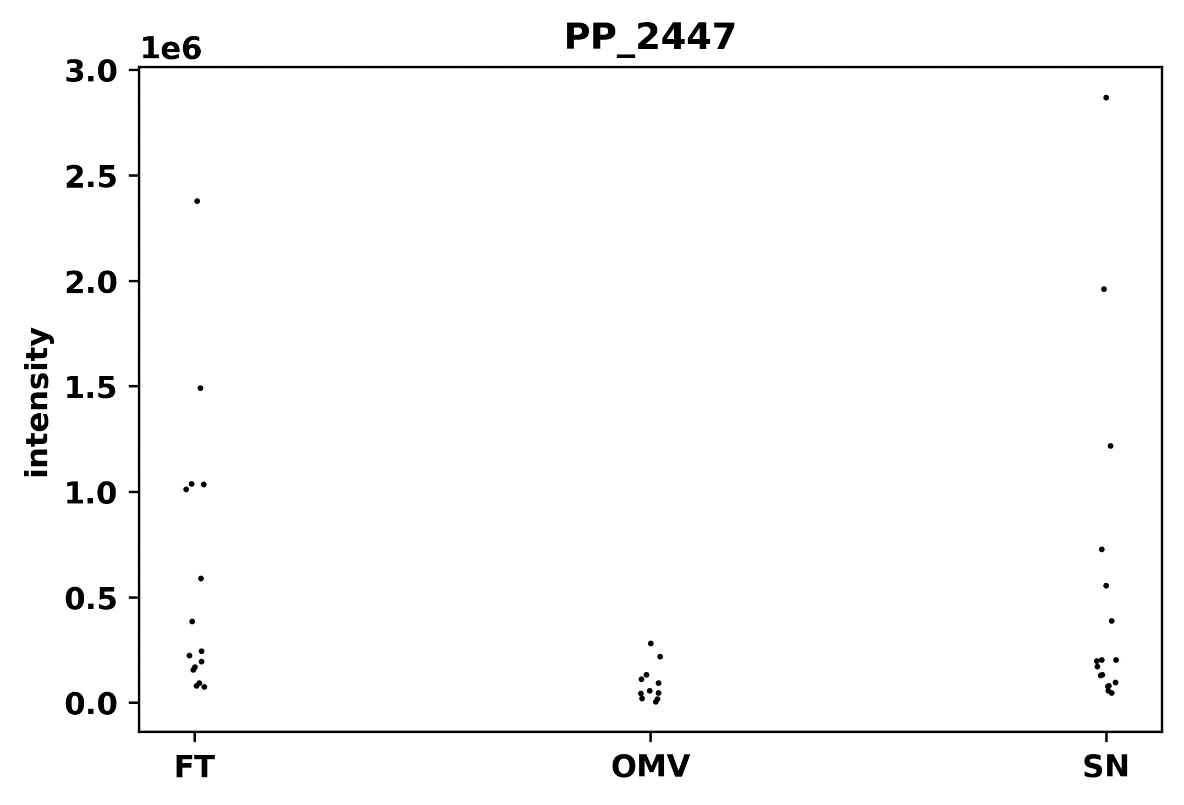

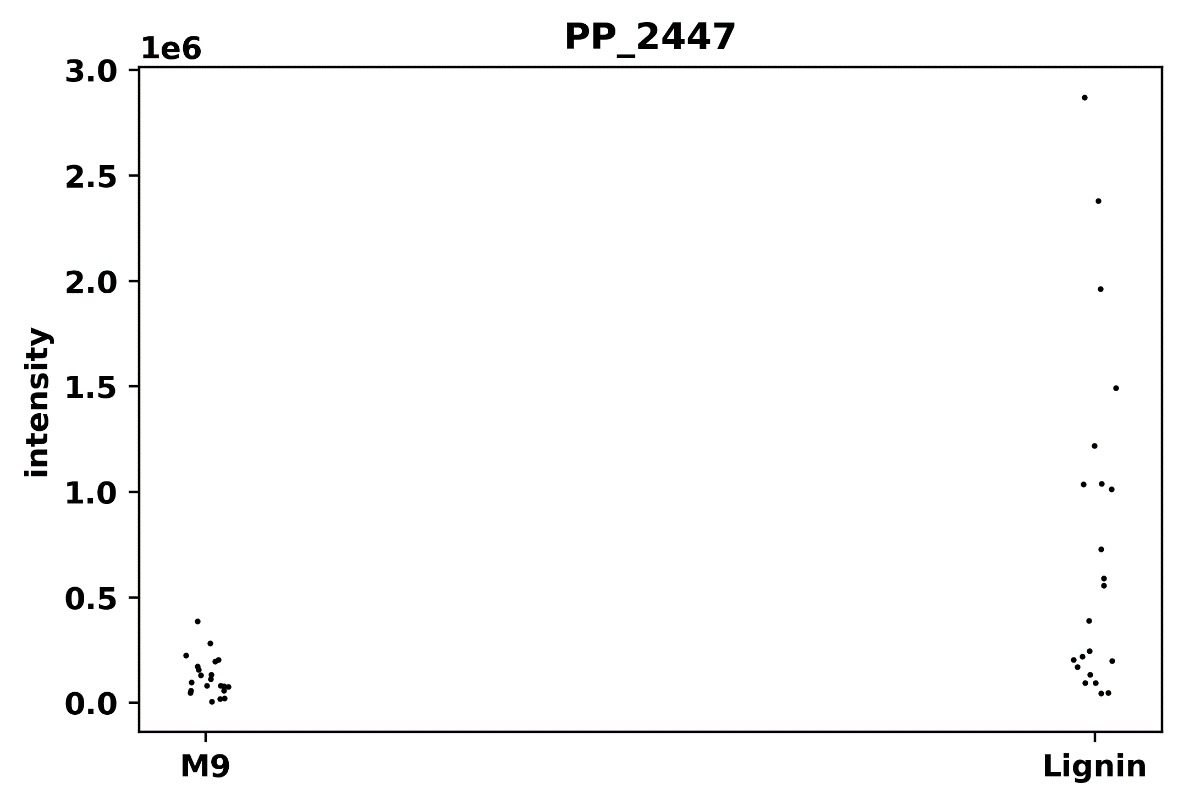


Notes:

The PUF’s identification in the supernatant is consistent with its predicted signal peptide. The first hit, Q8YY76 a Phytochelatin synthase-like protein from an unclassified Nostoc species (blue-green algae) has good structural homology except for a C-terminal pair of α-helices running parallel to each other linked by a loop. This seems to be an extension of the protein as the C-terminus in the hit protein ends before this section. This pair of helices are not found in any of the other hits either. Phytochelatin is a nonribosomal peptide heavy metal chelator produced by a broad array of eukaryotes. However, Phytochelatin synthases in prokaryotes seem to be much less active in the ‘synthesis’ bit of the process and may instead be peptidases, this is something of a controversy apparently. This hit seems to be a cystine peptidase. The authors of one of the papers presenting a structure for this hit hypothesize that this is part of an extracellular redox sensing system due to its reduced activity in the presence of oxidizing conditions and the fact that its product would be easy to sense.

A3DCU1 is another peptidase, this time one that is part of a peptidase containing ABC transporter complex from *C. therm*. It shares good structural homology with both PP_2447 and the previous hit.

P81297 is another cysteine proteinase, this time from *Staphylococcus aureus*. There’s little information about it that I could find but it again shares good homology with the previous proteins.

**PP_1726:**

The top structure hit was unpublished but listed as a putative ABC transporter, periplasmic substrate-binding protein from Campylobacter jejuni.

The second top hit (TM-score 0.83) is a Sugar ABC transporter, periplasmic sugar-binding protein from Thermus thermophilus

**PP_0891**:

predicted terms from structural homology model:

GO:0003824 catalytic activity

GO:0110165 cellular anatomical entity

GO:0005488 binding

top structure hits

TM-score: 0.98, Q48EL2 YceI-like family protein PSPPH_4050 Pseudomonas savastanoi pv. phaseolicola (strain 1448A / Race 6) (Pseudomonas syringae pv. phaseolicola (strain 1448A / Race 6))

TM-score: 0.97, Q21LI5 Cellulose-binding protein cbm2A Sde_1182 Saccharophagus degradans (strain 2-40 / ATCC 43961 / DSM 17024)

TM-score: 0.79, A2VST2 YceI BCPG_01022 Burkholderia cenocepacia PC184

TM-score: 0.79, P83815 YceI domain-containing protein nan Thermus thermophilus

TM-score: 0.79, Q9I690 UPF0312 protein PA0423 PA0423 Pseudomonas aeruginosa (strain ATCC 15692 / DSM 22644 / CIP 104116 / JCM 14847 / LMG 12228 / 1C / PRS 101 / PAO1)

interproscan results

"PP_0891","hypothetical protein","SUPERFAMILY","SSF101874","","IPR036761","21","191","1.7E-18","Lipid/polyisoprenoid-binding, YceI-like superfamily","5.36-75.0",""

"PP_0891","hypothetical protein","SMART","SM00867","","IPR007372","22","191","1.1E-13","Lipid/polyisoprenoid-binding, YceI-like","5.36-75.0",""

"PP_0891","hypothetical protein","Pfam","PF04264","YceI-like domain","IPR007372","23","166","5.2E-8","Lipid/polyisoprenoid-binding, YceI-like","5.36-75.0",""

"PP_0891","hypothetical protein","PIRSF","PIRSF029811","","IPR027016","1","193","1.8E-60","Uncharacterised protein with YceI domain","5.36-75.0",""

"PP_0891","hypothetical protein","Gene3D","G3DSA:2.40.128.110","","IPR036761","19","193","1.0E-46","Lipid/polyisoprenoid-binding, YceI-like superfamily","5.36-75.0",""

PP_0891 SignalP-5.0 signal_peptide 1 21 0.993590 . . .

proteomic evidence for existence: True

observed as differentially expressed: True


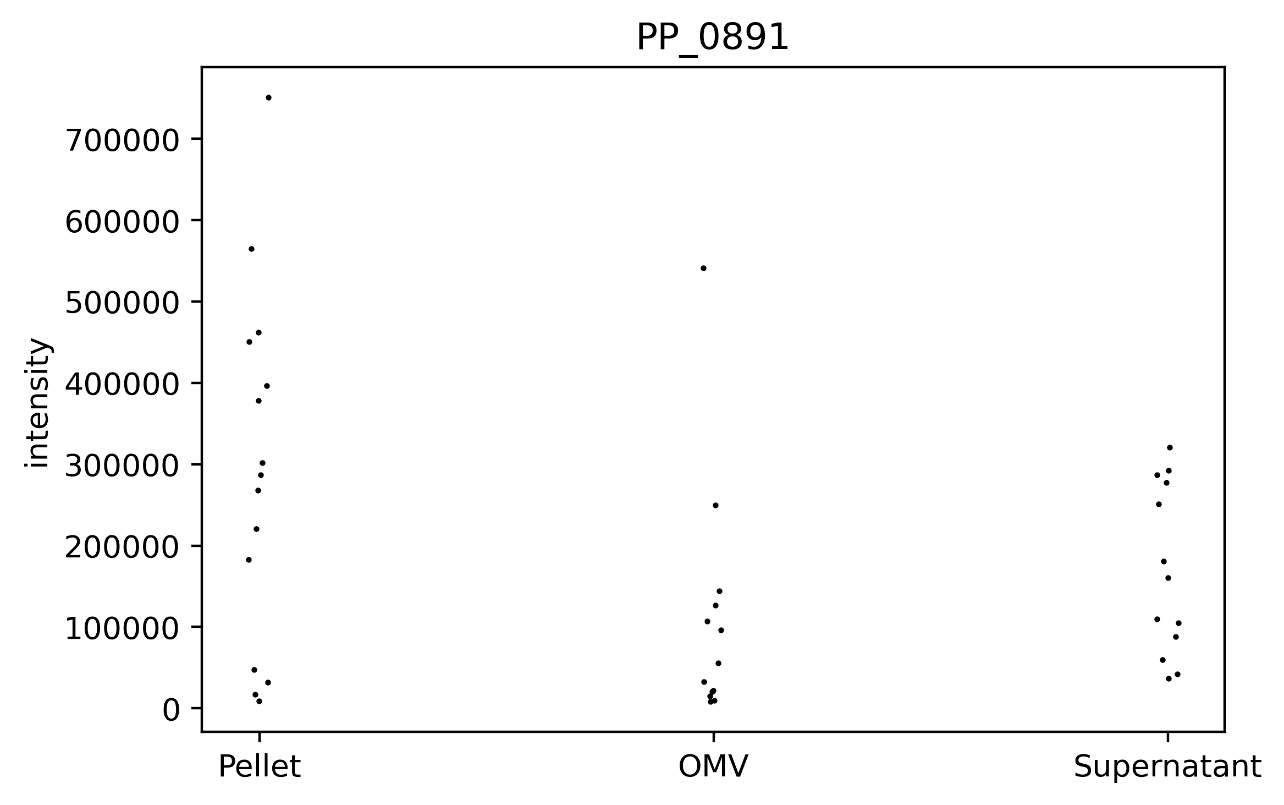

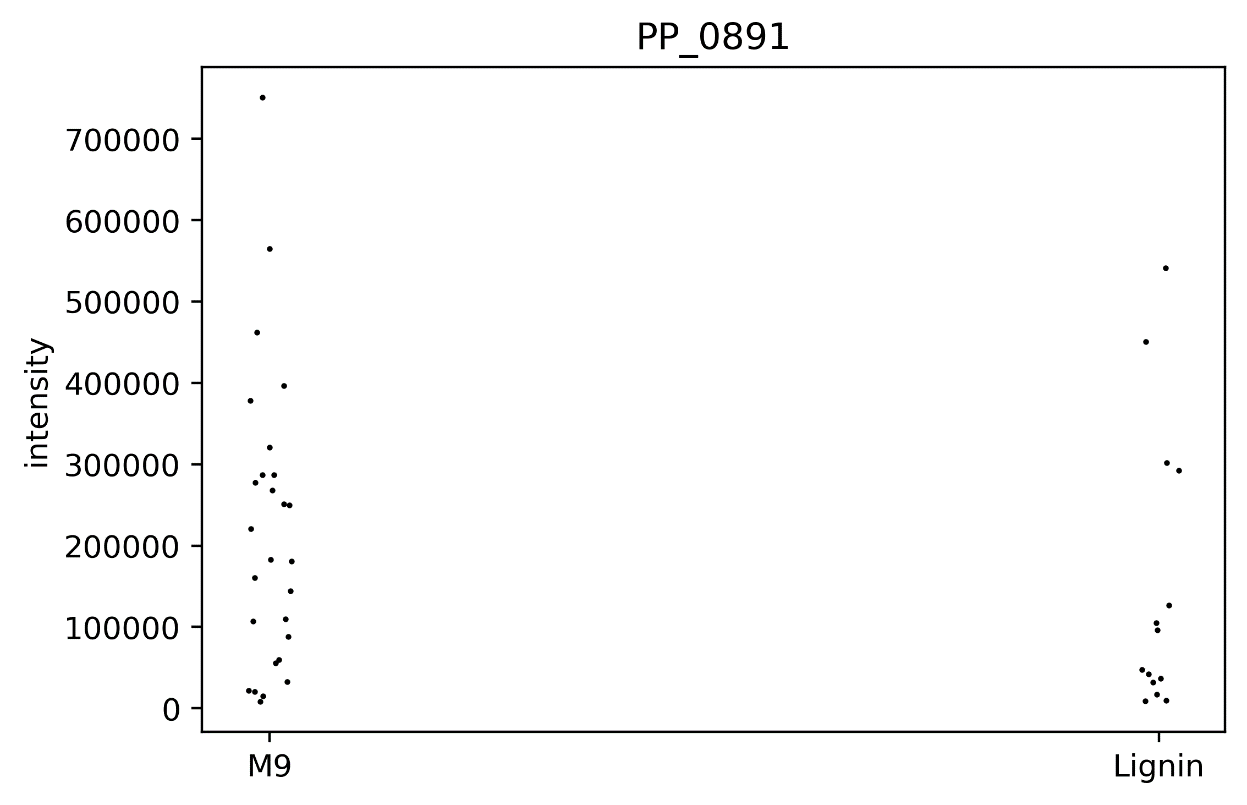


The top structure hit has no attached publication and the protein Q48EL2, a Ycel-like protein from Pseudomonas savastanoi pv. Phaseolicola has very little publicly available information about it. That said it’s expected to bind polyisoprenoids.

The second top structure hit, with a TM-score of 0.97, is also a Ycel-like protein this time from Saccharophagus degradans which also binds polyisoprenoids. This protein is implicated in plant cell wall breakdown. I think polyiosprenoid binding protein is a good annotation for PP_0891.

**PP_0576:**

Both signalP and interpro say it’s a lipoprotein. It shares moderate (TM-score ~0.55) similarity with an oligomeric PelC outer membrane polysaccharide export protein from *P. aeruginosa* which is a lipoprotein and a CsgG curli biogenesis system protein which is also a lipoprotein that forms an outer membrane pore in *E. coli*. The core of both of these proteins matches quite well with PP_0576 but the membrane spanning β-barrel domain doesn’t match well largely because it looks mispositioned. I’m willing to say this is an outer membrane pore forming protein although what it’s transporting is an open question that I don’t think structural data will be able to resolve. Of note, both proteins are involved in exporting biofilm constituents.

**PP_1397:**

The fragment is too small to get good hits

**PP_1726:**

Has signal peptide and is “ABC transporter substrate-binding protein” according to interproscan. Is also actually annotated.

**PP_2007:**

“P-47 like protein” P-47 is a protein from the botulin toxin operon in *C. botulinum* that does not have a known function. There is a very strong structural similarity between PP_2007 and P-47. P-47 has a tubular lipid binding (TULIP) fold which, as the name suggests, is used for binding lipids and other long hydrophobic substrates in some eukaryotes. A closely related protein from the same operon was experimentally shown to bind lipids in *C. botulinum*.

**PP_2306:**

Similarity to a protein involved in stabilizing the complement component C3 convertase complex in *Staphylococcus aureus* which is used as an immune evasion strategy. The hydrophobicities also match up to some degree so its reasonable to assume PP_2306 is doing something similar on a molecular level. Given that *P. putida* is not pathogenic its unlikely to be involved in the same biological process. It also has similarity to a hyperosmolarity resistance protein from *Staphylococcus epidermidis* but is missing a chunk. PP_2306 is pretty small so I’m not willing to say that these aren’t jus spurious hits. It’s probably a lipoprotein.

**PP_2363:**

CsuE like biofilm forming protein, pilin component. It has a really strong structural overlap with the exception of one domain that is also quite similar but the predicted relative positioning of this flexably linked domain is off.

**PP_2853:**

Has signal peptide, consensus disorder prediction and coiled coils prediction. The main structure of the protein is a β-barrel with an α-helix occluding one end. There’s a long, unstructured and predicted disordered region connecting to a freely positioned α-helix. Strong similarity to a polypeptide exporter protein (FapF) involved in the construction of amyloid biofilm components in *Pseudomonas sp. UK4*.

**PP_3350:**

Has predicted signal peptide and “alginate export domain”. Present strongly in pellet and supernatant but not OMVs, general upregulation in presence of lignin unlikely. The protein is a large β-barrel that matches strongly to Alginate biosynthesis protein AlgE from *P. aeruginosa*. This is also an exporter of biofilm constituents. PP_3350 has been previously identified by CBI as a low homology alginate porin relative that is important for *P. putida* tolerance of hydroxycinnamic acid derivatives. It also shares close structural similarity to multiple other porins with diverse substrate specificities.

**PP_3445:**

Bad structural matches, no other information.

**PP_3954:**

Interproscan says it’s a substrate binding protein and/or a TRAP transporter solute receptor, TAXI family. Has a signal peptide. High in supernatant and pellet low in OMV. Reliably upregulated in lignin. In the genome its right between trkH-I, a potassium transporter and PP_3955, a ‘permease’. It has very strong similarity to a putative periplasmic glutamate/glutamine-binding protein from *Thermus thermophilus* as well as a immunogenic protein from ehrlichia chaffeensis for which I cannot find molecular details of its behavior. This is probably a periplasmic substrate binding protein related to the neighboring permease.

**PP_3985:**

This was already annotated as a transposase

**PP_4906:**

Interproscan calls this a Metal-dependent hydrolase HDOD. And says it has coils. Its only getting terrible RUPEE hits

**PP_5363:**

Has signal peptide. RUPEE hits are too poor to be informative. Protein has a mostly complete β-barrel core

**PP_5531:**

High in OMVs but not pellet or supernatant, reliably downregulated in response to lignin. Closely structurally related to a number of other PUFs one of which is described as potentially being a metal-dependent hydrolase. Interproscan annotates it with "DinB/YfiT-like putative metalloenzymes"

**PP_5710:**

Lipopoprotein signal peptide from both interpro and singalp. Structural similarity hits are garbage though.

**PP_5737:**

No information from interproscan and structural hits max out at a TM-score of 0.43 which hits against a protein that binds methylated adenosine in the motif G(m^6^A)C. The overall shape of the proteins are the same but the fit it too sloppy for me to feel comfortable saying they have similar functions.
